# NiO@conducting polymer electrocatalyst for hydrazine-assisted oxygen evolution reaction through water splitting

**DOI:** 10.1038/s41598-025-09480-3

**Published:** 2025-07-25

**Authors:** Ekram H. El-Ads, Moshira M. Khalil, Mahmoud A. Abd El-Ghaffar, Ahmed Galal

**Affiliations:** 1 Faculty of Science, Chemistry Department, Cairo University, Giza, 12613 Egypt; 2 Polymers and Pigments Department, National Research Center, Cairo, Egypt; 3 Basic Science Department, October High Institute For Engineering & Technology (OHI), 6 October, Giza, Egypt

**Keywords:** Hydrazine-assisted water splitting, Electro-catalyst, Conducting polymers, Nickel oxide, Oxygen evolution reaction, Hydrogen production, Chemistry, Energy science and technology, Materials science, Nanoscience and technology

## Abstract

The energetic suitability of electrochemical water splitting is hindered by the sluggish kinetics of oxygen evolution reaction (OER). The electrochemical oxidation of hydrazine (HyzOR) preceding OER is a straightforward approach to generate H_2_ with lower overpotential and fast kinetics. Herein, a single-step synthesis approach for a nickel oxide nano-catalyst (NiO) mixed with a conducting polymers mixture of poly (m-toluidine) and poly (3,4-ethylenedioxythiophene) modified carbon paste electrode (CPE) is presented. Compared with CPE, NiO, and polymer mixture, the presented composite shows higher electrocatalytic activity for both HyzOR and OER in alkaline medium of KOH (HyzOR-OER). The presented composite exhibits a stable current response for hydrazine-assisted water splitting during exhaustive electrolysis. Also, it shows relatively low activation energy of 2.90 kJ.mol^− 1^ that reflects the facilitation of HyzOR-OER at this surface. The hydrazine-assisted water splitting by this surface is a committed feasible approach for low cost and high efficiency H_2_ production.

**Supplementary Information:**

The online version contains supplementary material available at 10.1038/s41598-025-09480-3.

## Introduction

Hydrogen, a renewable energy source, has been presented as an effective alternative to fossil fuels because of its high energy density and zero CO_2_ emission^1,2^. However, the sustainable and clean production of H_2_ is still a challenge. One of the principal strategies to address this challenge is green electrochemical water splitting^3–5^. Two main half-reactions have been involved in the electrochemical water splitting, namely, cathodic hydrogen evolution reaction (HER) and anodic oxygen evolution reaction (OER)^2,6,7^. The OER is considered a major challenge in electrochemical water splitting because it is a sluggish anodic reaction with high overpotential restricting the H_2_ generation^8–10^. To promote the H_2_ production via electrochemical water splitting, two major approaches can be considered, namely, design an efficient OER electrocatalyst^11–14^ or utilizing the oxidation of small active molecules as a “promoter” to direct OER^6,15–17^. Different small molecules have been employed and mentioned in literature such as methanol^18^, formaldehyde^19^, urea^20^, and hydrazine^1,6,7,15,16,21–23^.

Hydrazine is not abundant in nature, however its industrial production mounts to 30,000 metric tons per year or higher. The most well-known applications of this chemical are in agriculture, polymer industries, rocket propellants, and water treatment. Hydrazine oxidation is considered a viable source for enhancing the oxygen evolution reaction in alkaline media. Hydrazine in the context of the present work is used to achieve lower onset-/over- potential and higher reaction rates. Furthermore, hydrazine oxidation yields eco-friendly products and reacts steadily with a variety of electrocatalysts. OER is challenging in alkaline media; besides the relatively high overpotential required, the catalysts used are not cost effective and lack long term stability.

The sluggish anodic OER can be assisted by the thermodynamically favored hydrazine oxidation reaction (HyzOR) to enhance the HER, and this can be represented as (HyzOR-OER). This is attributed to the facile charge exchange kinetics, relatively high-energy density for the conversion process and lower oxidation potential of hydrazine^1,15,16,23,24^. The HyzOR-OER principle in alkaline medium is based on the assistance of anodic OER by HyzOR producing N_2_ gas at the anode and H_2_ gas at the cathode. This process is known as hydrazine-assisted HER^1,15^. The products of this process are much safer than O_2_ and H_2_ in the electrochemical water splitting. Therefore, hydrazine-assisted electrochemical water splitting is more effective, safe and economically desirable for green H_2_ production^15,16,23^.

On the other hand, developing electrocatalysts with high catalytic performance is essential to reduce the overall cell potential of water splitting^25–28^. Recently, transition metal oxides (Ni, Co, Fe, etc.) have presented good criteria such as electrochemical performance, affordability, stability and availability to be effective OER electrocatalysts^29–31^. Among them, NiO nanostructure is an efficient electrocatalyst, with high surface area, more available active sites and good stability, which can be tailored in different approaches to improve its electrocatalytic conductivity and activity as mentioned in literature^7–11,13,14^. Few papers in literature have mentioned the utilization of NiO based electrocatalyst for hydrazine-assisted water splitting^32–36^.

Conducting polymers such as polythiophene, polyacetylene, polypyrrole and polyaniline have unique properties enhancing their applications in different fields^37–39^. They have been widely utilized as catalyst support for the oxidation of small molecules, and they are permeable to electroactive species^40^. Polyaniline and its derivative (poly (m-toluidine), PMT) exhibit good optical, electrical and thermoelectric criteria^37,41^. PMT is a conjugated conducting polymer with a backbone consisting of two main groups of benzenoid group (electron-rich) and quinoid group (electron-deficient)^37^. PMT shows processability, relatively low cost, environmental stability, and good conductivity to be utilized in corrosion inhibition, catalysis, sensors, and batteries^37,40–44^.

On the other hand, poly (3,4-ethylenedioxythiophene) (PEDOT), a derivative of polythiophene, exhibits good characteristics of ease adjustment of molecular chain structure, ionic and electronic conductivity, photo and electrical conductivity, biocompatibility and stability^45–47^. PEDOT exhibits ease of oxidation and a good tendency toward multiple redox switching owing to its linear chains structure which blocks the 3,4-positions of thiophene ring^47^. Few polymer-based electrocatalysts for hydrazine-assisted water splitting have been cited in literature^48,49^.

In this work, we present a single-step procedure for a nickel oxide nano-catalyst (NiO) mixed with a conducting polymer mixture of poly (m-toluidine) and poly (3,4-ethylenedioxythiophene) modified carbon paste electrode (CPE). The combination of PMT with PEDOT with the aid of NiO has not been reported elsewhere in the literature for hydrazine-assisted water splitting. The proposed surface PMTPEDOTNiOCPE is employed for HyzOR-OER and proved highly competitive with those previously reported in the literature. A summary of the methodology and the objective of this work is shown in Supplement Fig. 1.

## Experimental

### Chemicals and materials

The chemicals used in this work are as follows: KOH (anhydrous, ≥ 99.95% trace metals basis), hydrazine hydrate (reagent grade, N_2_H_4_ 50–60%), NiO nano-powder (< 50 nm particles’ size: BET, 99.8% trace metals basis), m-Toluidine (99%), o-Toluidine (99%), 3,4-ethylenedioxythiophene (EDOT, 97%), acetonitrile, tetrabutylammonium hexafluorophosphate (TBAHFP, 98%), dimethylformamide (DMF), graphite powder, and paraffin oil. All chemicals are purchased from Sigma-Aldrich (USA) and used without further treatment.

### Preparation of different polymers

**Fig. 1:**
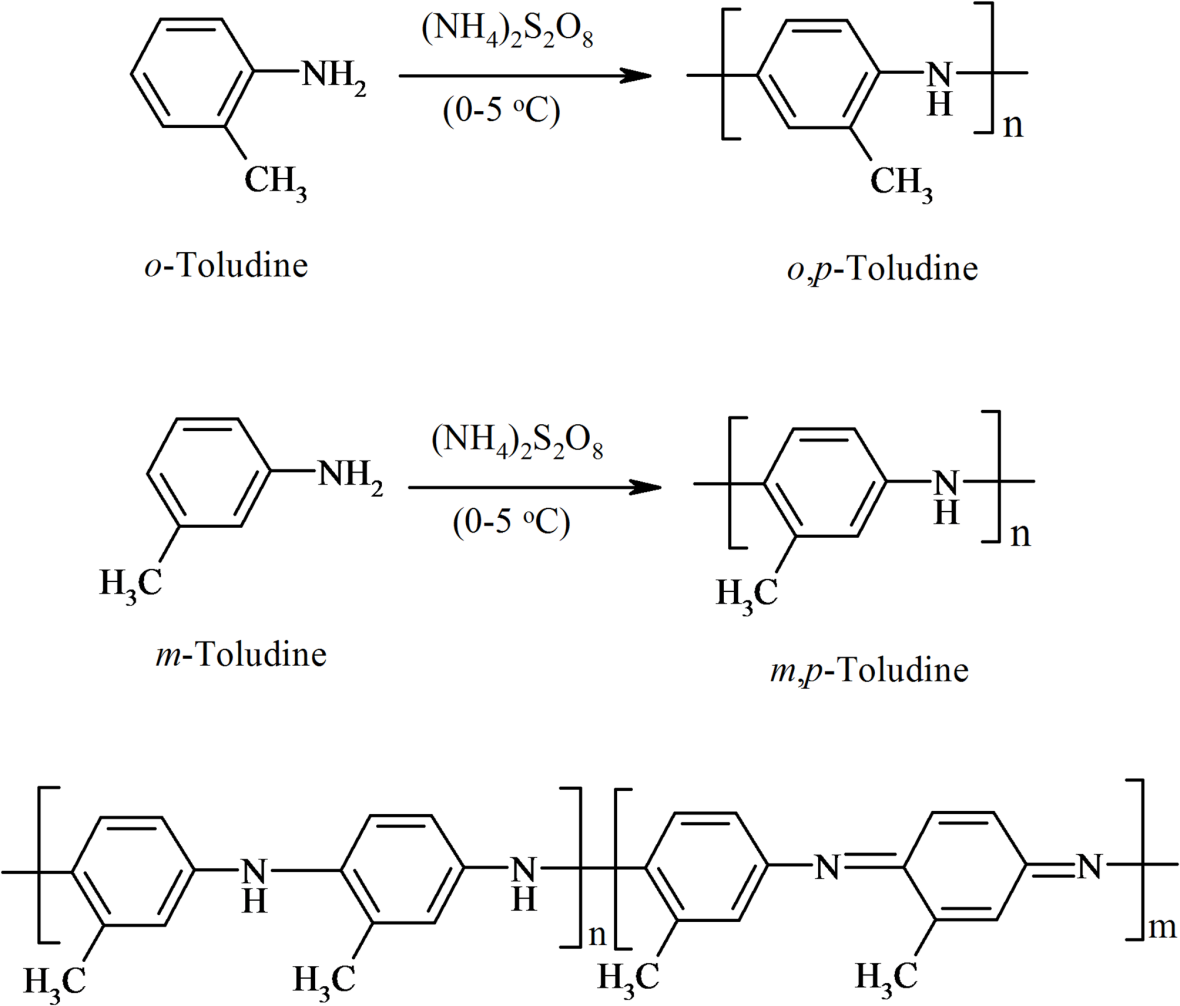
Preparation of poly (o-toluidine) and poly (m-toluidine).

Poly(m-toluidine); PMT and poly(o-toluidine); POT powders are prepared by chemical oxidation polymerization method. A 0.01 M solution of the respective monomer (prepared in absolute ethanol) is combined with ammonium peroxydisulfate (APS) as oxidant and 0.01 M HCl as dopant (the initial mixing ratio of monomer and HCl is about 1:1) for 20 h at 0–5 °C and stirring in an ice bath. The resulting precipitates are filtered and washed with hot double distilled water and ethanol to remove the oxidant present until the filtrate turned colorless, which is then dried at 60 °C for 24 h in a vacuum oven. The final powder obtained through the above steps is poly(m-toluidine) or poly(o-toluidine) depending on the monomer used^42,50^. Figure 1 summarizes the preparation method of both polymers.

Poly(3,4-ethylenedioxythiophene); PEDOT is electropolymerized on Pt sheet from 0.05 M EDOT/0.1 M TBAHFP in acetonitrile using bulk electrolysis by applying a constant potential of 1400 mV for 20 min^51^. The polymeric film is left to dry and scraped off the surface of Pt for further use. This step is repeated several times to obtain the required mass of PEDOT for further use.

To prepare a polymer mixture of poly (m-toluidine) and poly (3,4-ethylenedioxythiophene) PMTPEDOT; a series of PMTPEDOT is prepared by mixing PMT with different amounts of PEDOT (0.5%, 1%, 1.5% and 3%) and dissolved in DMF. The obtained polymer mixture is sonicated for about two hours till complete and homogenous distribution is achieved and then left to dry in air. Then the optimum ratio of PMTPEDOT is selected by measuring the electrical properties. Another polymer mixture of POTPEDOT is prepared by the same method using POT and 1.5% PEDOT for comparison.

### Measurement of electrical properties of polymers using LCR Bridge

Different electrical properties (Ohmic resistance, inductance, capacitance, etc.) are measured using LCR bridge circuit operated on AC (HIOKI 3532-50 LCR HITESTER) at the frequency range of 10^3^-10^6^ Hz. The electrical properties of a series of PMTPEDOT mixture (PMT mixed with different amounts of PEDOT: 0.5%, 1%, 1.5% and 3%) are measured to select the optimum ratio.

### Electrochemical instrumentation and cell

A PGZ301 Volta lab Galvanostat/Potentiostat (Radiometer Analytical, USA) is used to measure voltammetry and chronoamperometry in a three-electrodes cell setup: carbon paste electrode CPE (*φ*: 6.4 mm), Ag/AgCl (3.0 M KCl) and a Pt wire (*l*: 10 cm, *φ*: 2.0 mm) as working, reference and auxiliary electrodes, respectively. Linear sweep voltammetry curves are recorded from E_i_ = -1.2 V to E_f_ = 1.6 V at scan rate of 50 mV s^− 1^. The Tafel experiments are conducted at a scan rate of 1 mV s^− 1^. The chronoamperometry experiments are performed by applying a constant applied potential equivalent to the onset potential of HyzOR at a given catalyst surface for certain time. All potential values are normalized to RHE using the following formula: E(RHE) = E_Ag/AgCl_ + 0.059 pH + E^o^_Ag/AgCl_.

The theoretical potential for OER in water splitting under standard conditions is 1.23 V vs. RHE. The overpotential can be calculated using the following formula:

Overpotential = Onset potential − Theoretical potential (= 1.23 V).

### Electrode Preparation

Graphite powder (0.1 g) is mixed with 36 µL paraffin oil in a mortar and pestle to prepare CPE as mentioned earlier^52^. PMT modified with 1.5% PEDOT is chosen from electrical properties study, and it is represented as PMTPEDOT. Modified CPE is prepared by mixing 5% of PMTPEDOT with different amounts of NiO nanopowder (1, 3, 5, and 7%) and graphite powder to obtain 100% total percent of modified graphite. Paraffin oil (36 µL) is added to each 0.1 g of modified graphite and mixed in a mortar till a homogenous paste is obtained. These surfaces are tested in 0.5 M KOH to select the optimum percent of NiO nanopowder and found as 3%(figure not shown). The previous step was repeated with respect to PMTPEDOT to optimize its ratio with 3%NiO modified CPE. These surfaces are tested in 0.5 M KOH and the optimum percent of PMTPEDOT is 3%with 3%NiO (figure not shown). The optimized surface is named PMTPEDOTNiOCPE. The different surfaces are renamed using the Roman numbering as follows: (I) CPE, (II) NiOCPE, (III) PMTPEDOTCPE, (IV) PMTNiOCPE, (V) PEDOTNiOCPE, (VI) PMTPEDOTNiOCPE, and (VII) POTPEDOTNiOCPE.

All studied surfaces are tested in 0.5 M KOH and 0.5 M hydrazine/0.5 M KOH. All measurements are conducted at 25 ± 0.1 °C.

Real surface areas of the different proposed surfaces have been calculated from the data obtained by conducting cyclic voltammetry experiments (Supplement Fig. 2) in potassium ferricyanide (1.0 mM K_3_[Fe(CN)_6_] electrolyte) using Randles Ševćik equation as mentioned before^53^. The values of real electroactive surface areas are 0.206 cm^2^, 0.310 cm^2^, 0.557 cm^2^, 0.629 cm^2^, 0.446 cm^2^, 0.725 cm^2^ and 0.801 cm^2^ for I, II, III, IV, V, VI, and VII, respectively.

### Structural and surface analysis

Thermal gravimetric analysis (TGA) is performed using a Shimadzu TGA-50 H instrument in the temperature range from room temperature to 800 °C in air with a heating rate of 5 °C/min. This analysis is performed for different samples namely: NiO, PMTNiO, PEDOTNiO, PMTPEDOTNiO, and PMTPEDOTNiOCPE. FTIR is used for functional groups characterization using Shimadzu (Japan) IR-Affinity Spectrometer for NiO, PMTNiO, PEDOTNiO, PMTPEDOTNiO, and PMTPEDOTNiOCPE.

UV-vis measurements are performed for POT, PMT, PEDOT, PMTPEDOT and POTPEDOT at room temperature using a computerized recording on Cary 300 spectrophotometer, Agilent Technologies. The X-ray diffraction (XRD) spectra were recorded by Panlytical X’Pert using Cu-Kα radiation (λ = 1.540 Å).

The microstructure of PMTPEDOTNiOCPE, and PMTPEDOTNiO samples is investigated using field-emission scanning electron microscope (Quanta FEG 250 instruments). EDX coupled to SEM is used to analyze the composition of the prepared samples. High resolution transmission electron microscope (HR-TEM, Tecnai G20, FEI, Netherland, 200 kV, LaB6 Gun) is utilized to characterize the size and microstructure of the previous samples.

## Results and discussion

### Electrical properties of PMTPEDOT using LCR Bridge

Different electrical properties of a series of PMTPEDOT mixture (PMT with different amounts of PEDOT (0.5%, 1%, 1.5% and 3%)) are measured to select the optimum ratio. The electrical/electronic properties of the electrocatalyst materials are evaluated by using LCR meter. The first parameter is the electrical conductivity (*σ*/*G*) that reflects the ability of the material to conduct electricity in the solid-state setup. Supplement Fig. 3 (A and B) displays the effect of varying the applied frequency on the conductivity of poly (m-toluidine) (PMT) and the different physically mixed PMT with poly(3,4-ethylenedioxythiophene) (PEDOT). The general trend indicates first that PMT displays the lowest electrical conductivity over the studied frequency range; second, the highest conductivity is shown for the PMTPEDOT 1.5% mixture.

The electrical resistance in its two contexts, the polarization resistance (*R*_p_) and impedance (*Z*), clarifies the general performance for a given material when exposed to an applied potential. For this reason, electrical resistance is an important parameter when using the synthesized materials in contact with an electrolyte and subjected to an applied bias potential. The data shown in Supplement Fig. 3 (C and D) represents the variation of *R*_p_ and *Z* values with applied frequency, respectively. The behavior in both cases is practically the inverse of those displayed in Supplement Fig. 3 (A and B). Across the whole frequency domain, the physical mixture of PMT and PEDOT exhibits the lowest values for both *R*_p_ and *Z* values. The values for the mixture PMTPEDOT 1.5% are the lowest among all composites. The *Z* values reflect the “total” resistance when the material is subjected to an AC type electrical source.

The susceptance (*B*) is a measure of the susceptibility of a given material towards the change in current passing through it with time. The term (*B*) is related to the admittance (*Y*) and conductance (*G*) by the following relation (Eq. 1):

1 $$\:Y=G+jB$$

From the above equation, the real part is the conductance (*G*) while the imaginary part is the susceptance (*B*). The relation between the parameter (*B*) and the variation in applied frequency for studied materials is given in Supplement Fig. 3 (E). It is noticed that the loading of the mixture in the ratios PMTPEDOT 1.5% and 1.0% exhibited the highest values through the whole frequency range.

Therefore, for the aforementioned findings, we used the physical mixture PMTPEDOT 1.5% throughout the rest of this study while establishing comparison to other materials.

### Surface morphology and structural composition

#### SEM and TEM

**Fig. 2:**
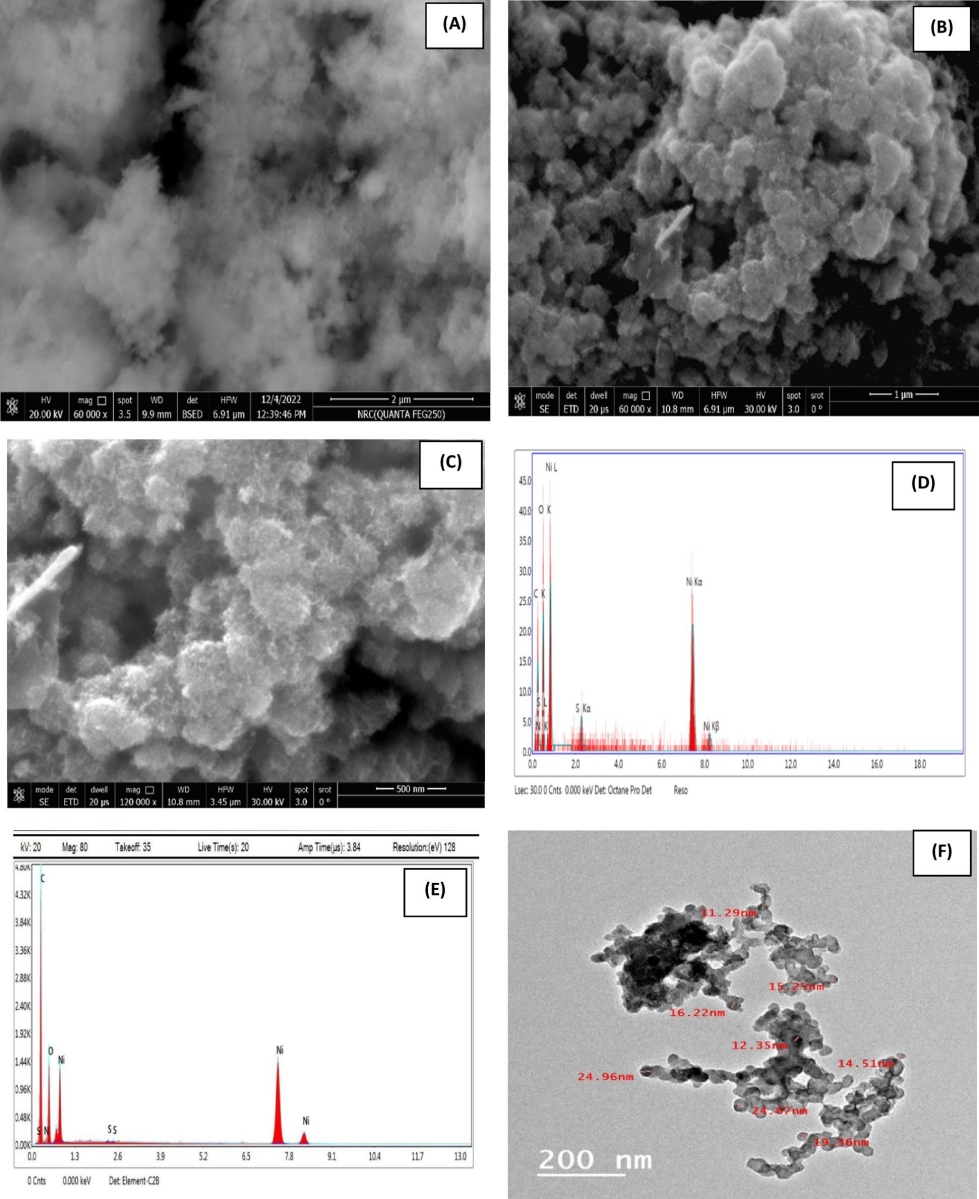

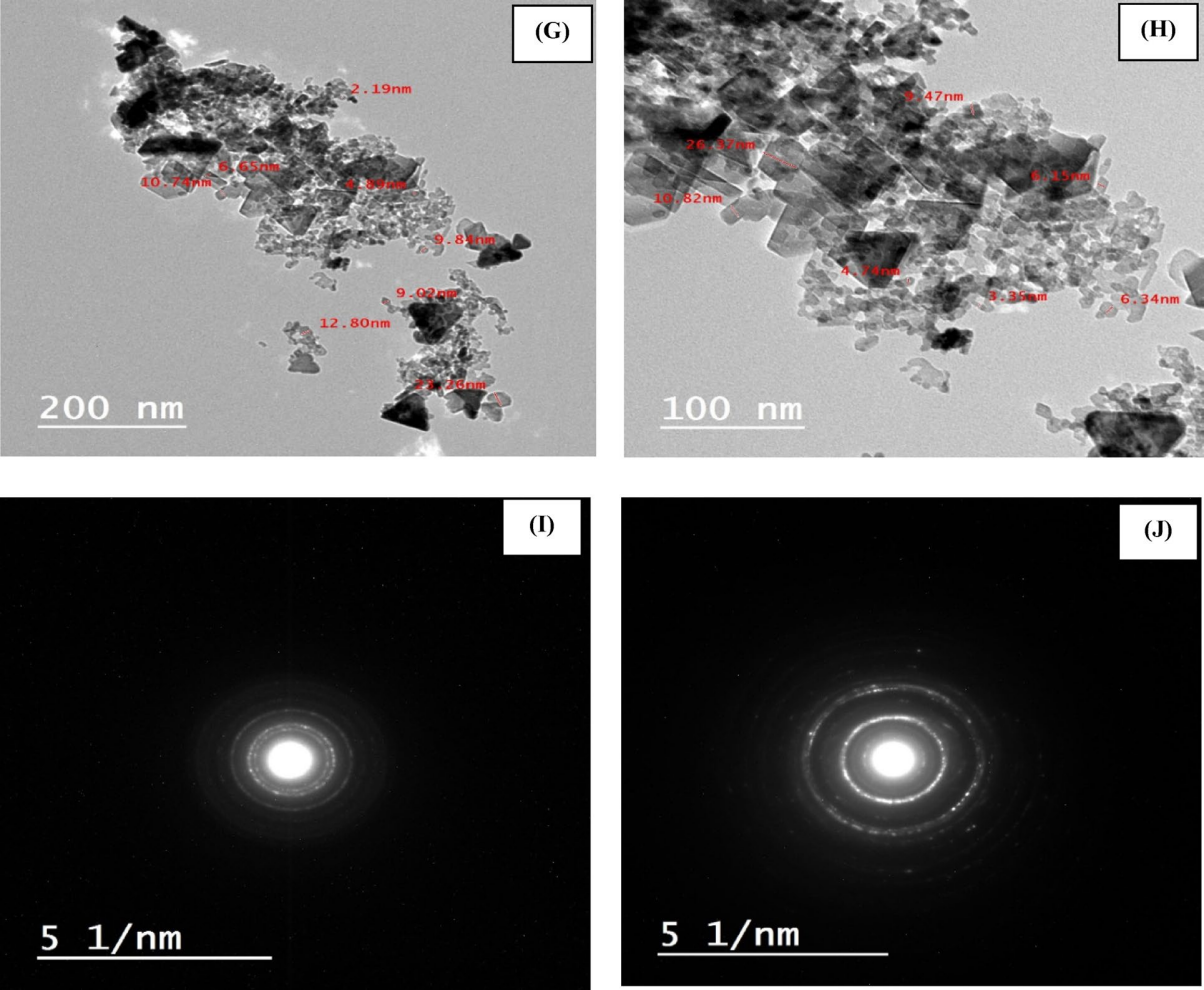
SEM of (**A**) PMTPEDOTNiO (60,000X), (**B**) PMTPEDOTNiOCPE (60,000X) and (**C**) PMTPEDOTNiOCPE (with higher magnification 120,000X). EDX of (**D**) PMTPEDOTNiO and (**E**) PMTPEDOTNiOCPE. HRTEM of (**F**) PMTPEDOTNiO (200 nm), (**G**) PMTPEDOTNiOCPE (200 nm) and (**H**) PMTPEDOTNiOCPE (with higher magnification 100 nm). SAED pattern of (**I**) PMTPEDOTNiO and (**J**) PMTPEDOTNiOCPE.

The morphologies of PMTPEDOTNiO and PMTPEDOTNiOCPE are investigated through SEM and TEM analyses. Figure 2 (A) shows the SEM of PMTPEDOTNiO, with NiO nanoparticles agglomerated over PMTPEDOT matrix. While Fig. 2 (B and C) shows the SEM of PMTPEDOTNiOCPE with different magnifications indicating the graphite flakes of carbon paste with NiO and PMTPEDOT matrix. The EDX of PMTPEDOTNiO and PMTPEDOTNiOCPE confirms the presence of all elements of the proposed composites with their expected ratios (Fig. 2 (D and E)), respectively. Figure 2 (F) shows the HRTEM of PMTPEDOTNiO with an average particles size of 15 nm of NiO. Figure 2 (G and H) shows the HRTEM of PMTPEDOTNiOCPE with different magnifications confirming the presence of graphite flakes with NiO (with an average particle size of 10 nm) assembled over polymer matrix. Figure 2 (I and J) shows the corresponding selected area electron diffraction (SAED) pattern of the TEM images of PMTPEDOTNiO and PMTPEDOTNiOCPE, respectively. The detailed HRTEM for PMTPEDOTNiO electrocatalyst is provided as Supplement Fig. 4 from which the SAED were extracted. HRTEM shows the textures of the structured materials and SAED is analyzed for the different phases of crystal structure. Also, the plane values were extracted mainly for NiO.

**Fig. 3:**
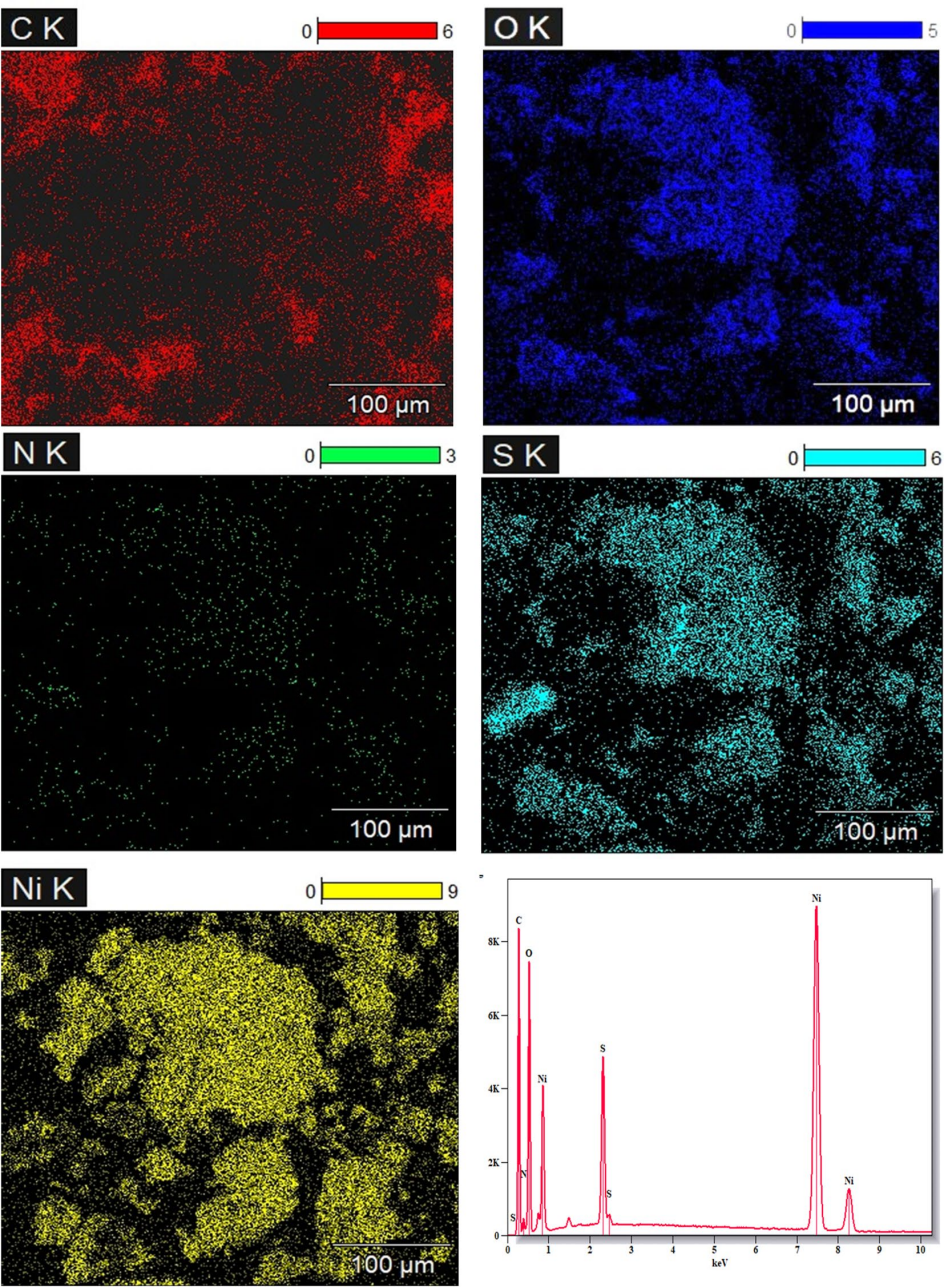
Elemental mapping of the surface of PMTPEDOTNiO electrocatalyst.

The data for the elemental mapping for the PMTPEDOTNiO electrocatalyst is provided in Fig. 3 to confirm the elemental distribution over the surface.

### UV, FTIR and TGA

Supplement Fig. 5 shows the UV-vis absorption spectra of POT, PMT, PEDOT, PMTPEDOT and POTPEDOT. POT shows only one absorption band at 270 nm. While PMT, PEDOT, POTPEDOT and PMTPEDOT exhibit good absorption spanning the UV-vis range with two absorption bands at nearly 275 nm and 555 nm. The bands at 275 nm and 555 nm are due to π-π* electronic transitions of benzene rings and polaronic transitions, respectively^37,41,54,55^.

The FTIR spectrum of PMT is studied in literature and the most characteristic features are as follows: bending vibration of C-H of benzene ring at range from 611 to 775 cm^− 1^, N-H bending in secondary amines at 1559 cm^− 1^ and symmetrical bending vibration in C-H of aliphatic CH_3_ group from 1404 to 1492 cm^− 1 42^. While the spectrum of PEDOT as mentioned in literature shows absorption bands at 863 cm^− 1^ due to the structure of thiophene ring (C-S-C), 1073 cm^− 1^ and 1125 cm^− 1^ due to the structure of ethylenedioxy ring (C-O-C), 1360 cm^− 1^ and 1505 cm^− 1^ due to the stretching vibrations of C = C and C-C of thiophene ring and 2920 cm^− 1^ due to C-H vibrations in the CH_2_ moieties^45,56^.

The FTIR spectrum of NiO, as depicted in Supplement Fig. 6 (A), displays a strong broad absorption band at 3430 cm^− 1^ owing to the stretching vibrations of O-H of surface hydroxyl groups. Also, the attribution of water molecules is shown as an absorption band at around 1380 cm^− 1^. A sharp band is observed at 450 cm^− 1^ confirming the formation of Ni-O bonds within the crystal lattice of NiO^57,58^. Supplement Fig. 6 (B, C, D, and E) shows the FTIR spectra of PMTNiO, PEDOTNiO, PMTPEDOTNiO and PMTPEDOTNiOCPE, respectively with the characteristic absorption bands of the individual components confirming their presence in the composite.

The variation of mass over time as a function of temperature can be analysed via TGA technique^59^. The TGA of NiO, PMTNiO, PEDOTNiO, PMTPEDOTNiO and PMTPEDOTNiOCPE is investigated (Supplement Fig. 7). The TGA of NiO shows a stable thermogram up to 800 °C. For PMTNiO, a smooth weight loss step starts at ~ 200 °C followed by a sharp weight loss starting at ~ 500 °C. For PEDOTNiO, a sharp weight loss step starts at ~ 200 °C followed by a smooth weight loss at ~ 400 °C. The TGA of PMTPEDOTNiO shows a smooth weight loss at ~ 120 °C followed by a sharp weight loss starting at ~ 350 °C. While PMTPEDOTNiOCPE shows a sharp weight loss step starting at ~ 650 °C.

In addition, XRD was performed to illustrate the purity and crystallinity of PMTPEDOTNiO. The XRD pattern and refinement results are depicted in Supplement Fig. 8. The matrix of the composite contains two polymeric phases: poly(m-toluidine) (PMT) and poly(3,4-ethylenedioxy thiophene) (PEDOT) beside the “nano” nickel oxide (NiO). We used the Rietveld refinement to rationalize some details regarding the structural information of the composite. Beside several parameters that can be elucidated on the structure, we used the refinement procedure to confirm the different phases present in the structure of PMTPEDOTNiO. The diffraction shows a complex pattern reflecting the composition of the mixed sample. The NiO characteristic patterns appear at the following angles (2*θ*): 37.2°, 43.4°, 63.7°, 75.3° and 79.1° corresponding to the following planes: (111), (200), (220), (311) and (222), respectively. As indicated in the experimental section, NiO particles were dispersed over the two physically mixed polymers. The broadness of the peaks indicates that the particle sizes are below the micrometer range as expected. The diffraction angles region between 8° and 35° represents the diffraction of the two polymeric phases. PEDOT is semi-crystalline in nature and contains amorphous and crystalline regions. The degree of crystalline regions depends on the method of preparation of the polymer and the nature/degree of dopants. The optimized refinement of some identified diffraction peaks is labeled by asterisks (*) for PEDOT. PMT is comparable to poly(aniline) in its structure with the exception of the presence of the methyl group in the meta-position of the aromatic ring. The degree of crystalline nature of PMT depends also on the method of synthesis and nature/degree of dopant. However, PMT contains less regions of crystalline nature compared to PEDOT which is reflected in its low conductivity. Therefore, the region under consideration represents predominantly the diffraction due to PEDOT. The diffraction peaks denoted by “#” are characteristics of non-identified phase(s).

### Electrocatalytic activity of PMTPEDOTNiOCPE towards hydrazine-assisted water splitting process

#### Effect of hydrazine on the electrochemical behavior of PMTPEDOTNiOCPE electrode in basic electrolyte

**Fig. 4:**
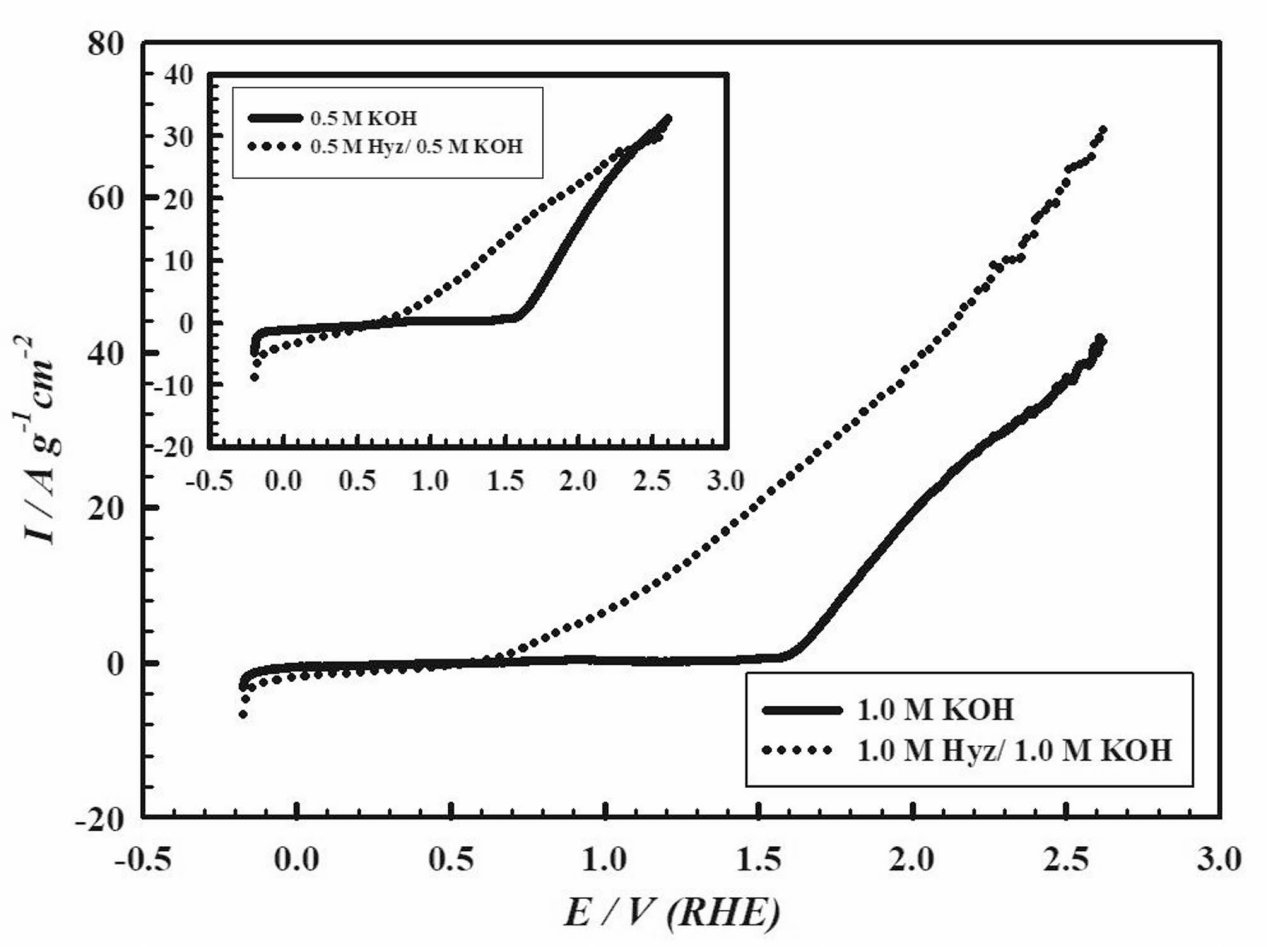
Linear sweep voltammogram (LSV) curves of electrode VI (PMTPEDOTNiOCPE) in 1.0 M KOH in presence and absence of 1.0 M Hyz. The inset: the same study in 0.5 M KOH in presence and absence of 0.5 M Hyz. Scan rate 50 mV.s^− 1^.

The electrocatalytic activity of electrode VI (PMTPEDOTNiOCPE) is assessed for oxygen evolution reaction (OER) in 1.0 M KOH in absence and presence of 1.0 M hydrazine (Hyz). Figure 4 displays the linear sweep voltammogram (LSV) curves of electrode VI in 1.0 M KOH in presence and absence of 1.0 M Hyz. The inset of Fig. 4 shows the same study in 0.5 M KOH in absence and presence of 0.5 M Hyz. The OER takes place in 1.0 M KOH at an onset potential of + 0.626 V (overpotential= ‒0.604 V) (RHE) in presence of 1.0 M Hyz with a specific current density of 0.903 A.g^− 1^.cm^− 2^. While the OER onset potential in absence of Hyz is + 1.605 V (overpotential = + 0.375 V) (RHE) with a specific current density of 0.380 A.g^− 1^.cm^− 2^. A shift in the potential of 0.979 V to more negative value is observed in 1.0 M Hyz/1.0 M KOH compared to Hyz-free solution (KOH only). These observations ensure that the presence of Hyz facilitates the OER owing to the ease electro-oxidation of Hyz in comparison to water. Each component of the presented surface, electrode VI, participates in the enhancement of the OER through their intrinsic characteristics as will be described later. The electro-oxidation of Hyz (N_2_H_4_) in basic medium is assumed to proceed according to the following mechanism producing hydrogen and ammonia (Eqs. 2–6)^60^:

2 $${{\text{N}}_{\text{2}}}{{\text{H}}_{\text{4}}}\,+\,{\text{4O}}{{\text{H}}^-} \to {\text{ }}{{\text{N}}_{\text{2}}}\,+\,{\text{4}}{{\text{H}}_{\text{2}}}{\text{O}}\,+\,{\text{4}}{{\text{e}}^-}$$

3 $${{\text{N}}_{\text{2}}}{{\text{H}}_{\text{4}}}\,+\,x{\text{O}}{{\text{H}}^-} \to {\text{ }}{{\text{N}}_{\text{2}}}+{\text{ }}({\text{4}}-x)/{\text{2}}{{\text{H}}_{\text{2}}}\,+\,x{{\text{H}}_{\text{2}}}{\text{O}}\,+\,x{{\text{e}}^-}$$

4 $${{\text{N}}_{\text{2}}}{{\text{H}}_{\text{4}}}\,+\,{\text{O}}{{\text{H}}^-} \to {\text{ 1}}/{\text{2}}{{\text{N}}_{\text{2}}}\,+\,{\text{N}}{{\text{H}}_{\text{3}}}\,+\,{{\text{H}}_{\text{2}}}{\text{O}}\,+\,{{\text{e}}^-}$$

5 $${{\text{N}}_{\text{2}}}{{\text{H}}_{\text{4}}} \to {\text{ }}{{\text{N}}_{\text{2}}}\,+\,{\text{2}}{{\text{H}}_{\text{2}}}$$

6 $${\text{3}}{{\text{N}}_{\text{2}}}{{\text{H}}_{\text{4}}} \to {\text{ }}{{\text{N}}_{\text{2}}}\,+\,{\text{4N}}{{\text{H}}_{\text{3}}}$$

In summary, to drive the same specific current density of 4.0 A.g^− 1^.cm^− 2^, the potential is set at + 0.80 V in presence and at + 1.71 V in absence of 1.0 M Hyz.

#### Scan rate effect

**Fig. 5:**
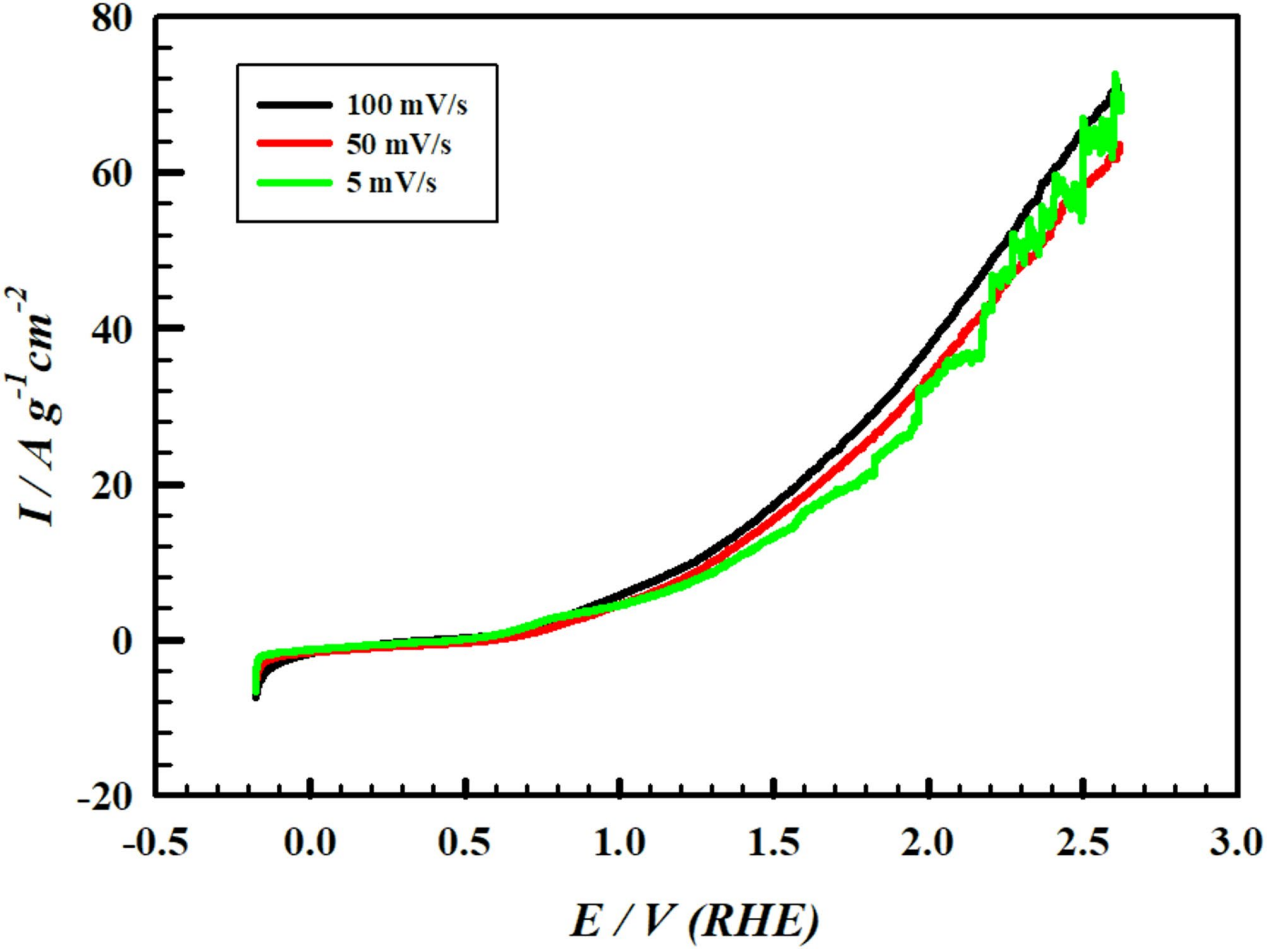
LSV curves of electrode VI (PMTPEDOTNiOCPE) in 1.0 M Hyz/1.0 M KOH at different scan rate values (5, 50 and 100 mV s^− 1^).

The scan rate effect on the OER is examined by studying the LSVs for electrode VI in 1.0 M Hyz/1.0 M KOH at different scan rates (5 to 100 mV s^− 1^). As the scan rate increases from 5 to 100 mV s^− 1^, a slight decrease in the onset potential values of HyzOR with a slight increase in the slopes of the LSV after the onset potential are observed (Fig. 5). The previous observations suggest effective charge and mass transfer during the water splitting process^61^. As the scan rate decreases, the rate of bubble formation decreases allowing large bubble formation and upon its departure from the surface, current hysteresis are noted.

#### Comparison of the electrochemical behavior of different surfaces in presence of hydrazine

**Fig. 6:**
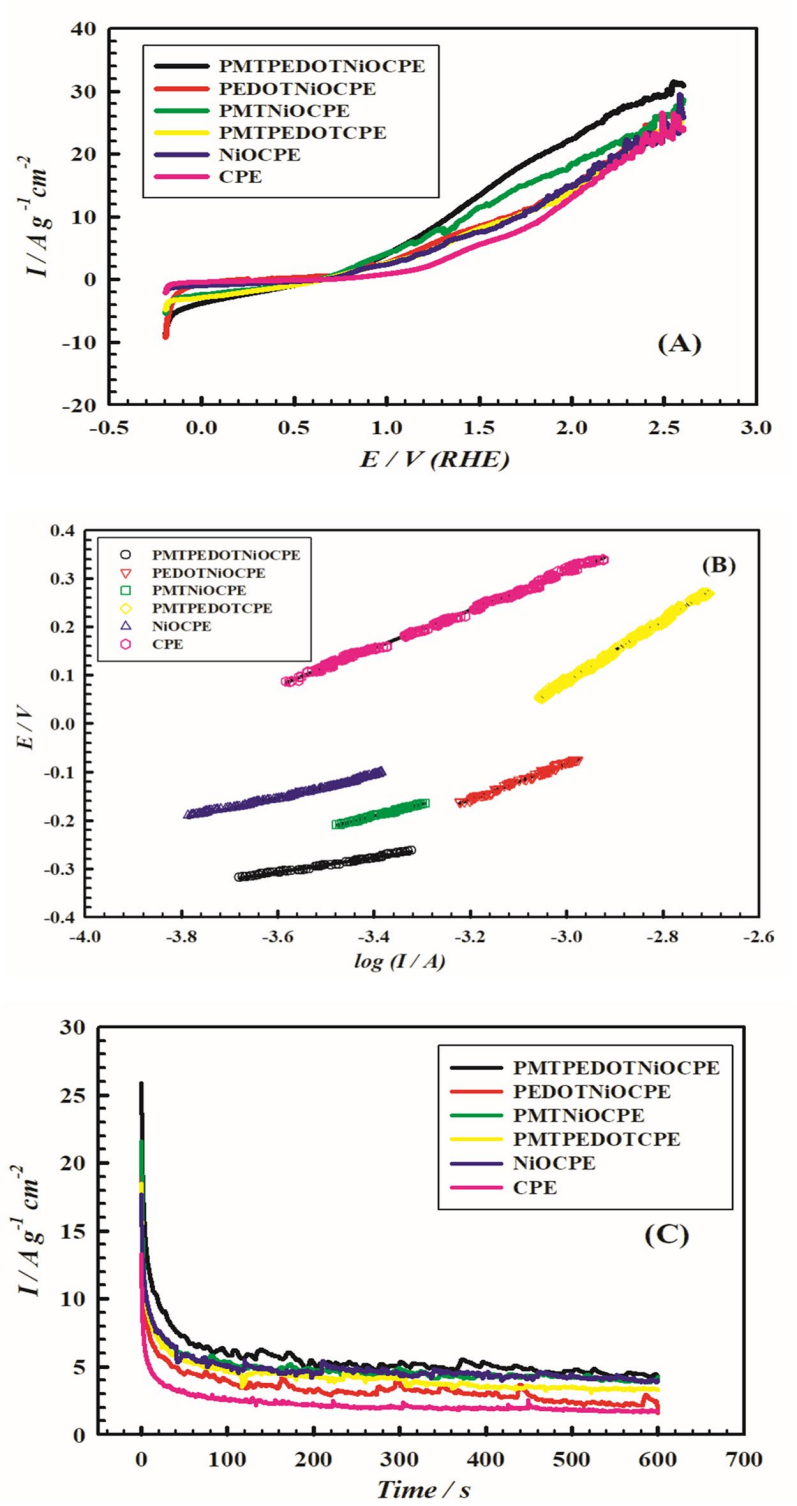
(**A**) LSV curves of different electrode surfaces in 0.5 M Hyz/ 0.5 M KOH, Scan rate 50 mV s^− 1^. (**B**) Tafel relations for HyzOR-OER at different electrodes in 0.5 M Hyz/0.5 M KOH; scan rate 1.0 mV s^− 1^. (**C**) Chronoamperograms over different electrode surfaces in 0.5 M Hyz/ 0.5 M KOH. Applied potential is 630 mV. (I) CPE, (II) NiOCPE, (III) PMTPEDOTCPE, (IV) PMTNiOCPE, (V) PEDOTNiOCPE, and (VI) PMTPEDOTNiOCPE.

The electrocatalytic activity of the different components of the present modified surface is compared. Figure 6 (A) shows the LSVs of electrode VI (PMTPEDOTNiOCPE), electrode V (PEDOTNiOCPE), electrode IV (PMTNiOCPE), electrode III (PMTPEDOTCPE), electrode II (NiOCPE) and electrode I (CPE) in 0.5 M Hyz/0.5 M KOH. The same study is conducted for the previously studied surfaces in absence of Hyz to estimate the influence of Hyz on the onset potential and rate of OER. Supplement Fig. 9 shows the LSVs in 0.5 M KOH in absence and presence of 0.5 M Hyz at electrodes V, IV, III, II, and I.

A prominent difference in the onset potential values for OER is observed upon the addition of Hyz. The onset potential value for Hyz-assisted OER is (+ 0.722 V ± 0.01 V) (overpotential= ‒0.508 V) for electrode VI compared to (+ 1.62 V ± 0.01 V) (overpotential = + 0.390 V) in Hyz-free medium (RHE). The onset potential value (vs. RHE) for Hyz-assisted OER is (+ 0.892 V ± 0.01 V) (overpotential= ‒0.338 V) for electrode V, (+ 0.752 V ± 0.01 V) (overpotential= ‒ 0.478 V) for electrode IV, (+ 0.87 V ± 0.01 V) (overpotential= ‒0.360 V) for electrode III, (+ 0.818 V ± 0.01 V) (overpotential= ‒0.412 V) for electrode II, and (+ 1.23 V ± 0.01 V) (overpotential = 0.0 V) for electrode I. While the values (vs. RHE) in Hyz-free medium for the previously mentioned surfaces are (+ 1.63 V ± 0.01 V) (overpotential = + 0.40 V), (+ 1.65 V ± 0.01 V) (overpotential = + 0.42 V), (+ 1.74 V ± 0.01 V) (overpotential = + 0.51 V), (+ 1.64 V ± 0.01 V) (overpotential = + 0.41 V), and (+ 1.77 V ± 0.01 V) (overpotential = + 0.54 V), respectively.

The overall water splitting process in basic medium is boosted upon using NiO nanoparticles^8,9^. Electrode VI manifests the best electrocatalytic performance for OER in absence and presence of Hyz. NiO nanostructures can reinforce the modified surface performance towards HyzOR-OER through its active surface area, available active sites and good electrical conductivity^7–11^. PMT is a conjugated conducting polymer with a backbone consisting of two main groups of benzenoid structure (electron-rich) and quinoid structure (electron-deficient)^37^. This structure can explain its interaction with PEDOT upon mixing. The combination of PEDOT with PMT and further NiO modification results in enhanced response towards electrochemical water splitting.

For all the studied surfaces, HyzOR-OER successfully precedes the OER which is the rate determining step in the water splitting process with a view to facilitate the hydrogen production (HER). In addition, the overpotential needed for HER is reduced in the presence of Hyz by reducing the overpotential needed for OER. The anodic and cathodic half-reactions and the overall reaction are suggested as mentioned in literature for single-atom electrocatalytic hydrogen production in presence of Hyz (Eqs. 7–9)^62^:

7 $${{\text{N}}_{\text{2}}}{{\text{H}}_{\text{4}}}\,+\,{\text{4O}}{{\text{H}}^-} \to {\text{ }}{{\text{N}}_{\text{2}}}\,+\,{\text{4}}{{\text{H}}_{\text{2}}}{\text{O}}\,+\,{\text{4}}{{\text{e}}^-}\left( {{\text{At the anode}}} \right)$$

8 $${\text{4}}{{\text{H}}_{\text{2}}}{\text{O}}\,+\,{\text{4}}{{\text{e}}^-} \to {\text{ 2}}{{\text{H}}_{\text{2}}}\,+\,{\text{4O}}{{\text{H}}^-}\left( {{\text{At the cathode}}} \right)$$

9 $${{\text{N}}_{\text{2}}}{{\text{H}}_{\text{4}}} \to {\text{ }}{{\text{N}}_{\text{2}}}\,+\,{\text{2}}{{\text{H}}_{\text{2}}}\left( {{\text{Overall reaction}}} \right)$$

The variation in the electrochemical performances of the different surfaces is probed by comparing their Tafel behaviors. The Tafel experiments are done for the following electrodes: VI, V, IV, III, II, and I in 0.5 M Hyz/0.5 M KOH at scan rate 1.0 mV.s^− 1^. These experiments are done by shifting the potential between + 50 mV to + 150 mV from the quasi-reversible onset potential regions for the OER for each surface. Tafel plots are shown in Fig. 6 (B). The Tafel slopes assumed from Fig. 6 (B) for the above-mentioned surfaces are 155, 370, 248, 629, 219 and 391 mV.dec^− 1^, respectively. The Tafel slope values are relatively higher compared to those previously mentioned in literature and other water splitting electrocatalysts^63,64^. The data suggests the ease charge transfer that denotes kinetically preferable routes for OER in the studied potential regions. Tafel slopes for the anodic reaction of OER are assessed from the d*E*/dln*j*_a_ ratio. The charge transfer coefficient α_a_ can be assessed from the following equation (Eq. 10) ^65^:

10 $$\:{(1-\propto\:}_{a})=\left(\frac{RT}{nF}\right)\left(\frac{d\text{ln}{j}_{a}}{dE}\right)$$

Where *n* is the number of electrons transferred in the oxidation reaction, *R* is the universal gas constant, *F* is Faraday’s constant and *T* is the absolute temperature. The charge transfer coefficients (α_a_) and the exchange current densities (*J*_°_) in 0.5 M Hyz/0.5 M KOH for OER for the studied surfaces are summarized in Table 1.

The values of the charge transfer coefficients for the HyzOR-OER for the surfaces VI, V, IV, III, II, and I in 0.5 M Hyz/0.5 M KOH are as: 0.905, 0.960, 0.940, 0.976, 0.933 and 0.962, respectively. The corresponding exchange current densities are as: 7.48 × 10^− 2^, 5.22 × 10^− 3^, 7.17 × 10^− 3^, 2.25 × 10^− 3^, 3.88 × 10^− 3^ and 4.88 × 10^− 4^ A.cm^− 2^, respectively for the corresponding surfaces. The reported values of charge transfer coefficients are comparatively higher compared to other processes mentioned in literature^66^. This may be interpreted in terms of irreversibility and complication of OER as indicated in equations number (2–6). Electrode VI shows higher value of exchange current density compared to other surfaces indicating the synergistic impact of the different components of the presented surface. This may result in facilitating the OER kinetics and reducing the corresponding energy barrier.

The apparent diffusion coefficient (*D*) and the rate constant (*k*) values of OER for the different electrode surfaces are estimated from chronoamperometry experiments. Figure 6 (C) shows the chronoamperograms for the different electrode surfaces in 0.5 M Hyz/0.5 M KOH. Constant potential values are applied and determined from the corresponding onset potentials of OER. A fast depletion of current with time is observed in the first few seconds reaching a semi-steady value after 100 s limit. Considerable variations within the first few seconds occur at the surface of the electrode affecting different kinetic parameters. *D* and *k* are calculated from the following equations (Eqs. 11, 12)^20^:

11 $$\:{I}_{p}=nFA{C}_{^\circ\:}{D}^{1/2}{\left(\pi\:\right)}^{-1/2}{\left(t\right)}^{-1/2}$$

12 $$\:{I}_{C}/{I}_{L}={\pi\:}^{1/2}{\left(kct\right)}^{1/2}$$

Where; *I*_p_ is the oxidation current of the electrocatalysis process (A), *C*_°_ is the Hyz concentration (0.0005 mol/cm^3^), *A* is the apparent electrode area (0.322 cm^2^), *n* is the number of electrons exchanged in the conversion process (*n* = 4), *F* is the Faraday’s constant (96486 C.mol^− 1^), *D* is the apparent diffusion coefficient (cm^2^.s^− 1^), *t* is the time (s), *I*_*L*_ is the current density value in absence of Hyz (KOH only), *I*_*C*_ is the current density value in presence of Hyz, and *k* is the catalytic rate constant (cm^3^.mol^− 1^.s^− 1^). Table 2 summarizes the values of *D*, and *k* for HyzOR-OER in presence and absence of 0.5 M Hyz in 0.5 M KOH for different surfaces. Electrode VI shows higher values of *D* and *k* compared to other surfaces indicating the synergistic impact of the different modifiers of the presented surface.

The diffusion coefficient is insightful to reflect the speed with which a charged molecule, ion or electron merge through the solution to reach the electrode surface. A more favorable mechanism reflects the effectiveness of the electrocatalyst for a given chemical conversion. When the number of charged species reaching the electrode surface increases, high diffusion coefficients are expected. Consequently, the reaction rate increases as the overpotential (the thermodynamic limitation) decreases. Besides the intrinsic properties of the components of the electrocatalyst film, a crucial factor is the electroactive surface area that contributes to the enhanced rate. When NiO nanoparticles (which are known electrocatalyst for OER) are added to the carbon paste, the rate constant increases. The inclusion of the conducting polymers PMT and or PEDOT increases the electrical conductance of the electrocatalyst and consequently enhances the rate of the reaction. Employing the complete components of the electrocatalyst, electrode VI, considerable increase in both the diffusion coefficient and the rate constant are observed. It is important to notice that the electroactive surface area increases progressively upon the addition of the components of the electrocatalyst. Also, the presence of NiO nanoparticles increases the concentration of the reacting species at the surface of the electrode.

**Table 1:** Exchange current density and charge transfer coefficient calculated from Tafel plots for different surfaces in 0.5 M Hyz/0.5 M KOH.

Surface	Charge transfer coefficient α_a_	Exchange current density j_o_ (A cm^− 2^)
VI (PMTPEDOTNiOCPE)	0.905	7.48 × 10^− 2^
V (PEDOTNiOCPE)	0.960	5.22 × 10^− 3^
IV (PMTNiOCPE)	0.940	7.17 × 10^− 3^
III (PMTPEDOTCPE)	0.976	2.25 × 10^− 3^
II (NiOCPE)	0.933	3.88 × 10^− 3^
I (CPE)	0.962	4.88 × 10^− 4^

**Table 2:** The diffusion coefficient, *D*, and catalytic rate constant, *k*, for HyzOR-OER in presence and absence of 0.5 M Hyz in 0.5 M KOH over different electrode surfaces.

Surface	0.5 M Hyz/ 0.5 M KOH		0.5 M KOH
	D / cm^2^.s^− 1^	k / cm^3^.mol^− 1^.s^− 1^	D / cm^2^.s^− 1^
VI (PMTPEDOTNiOCPE)	8.13 × 10^− 8^	1.05 × 10^4^	2.09 × 10^− 9^
V (PEDOTNiOCPE)	6.56 × 10^− 8^	2.20 × 10^3^	1.72 × 10^− 9^
IV (PMTNiOCPE)	6.72 × 10^− 8^	6.56 × 10^3^	1.85 × 10^− 9^
III (PMTPEDOTCPE)	2.90 × 10^− 8^	2.18 × 10^3^	5.84 × 10^− 10^
II (NiOCPE)	2.17 × 10^− 8^	1.95 × 10^3^	8.71 × 10^− 11^
I (CPE)	2.03 × 10^− 8^	1.04 × 10^3^	7.47 × 10^− 11^

#### Effect of concentration of KOH and hydrazine on the electrochemical water splitting at PMTPEDOTNiOCPE

**Fig. 7:**
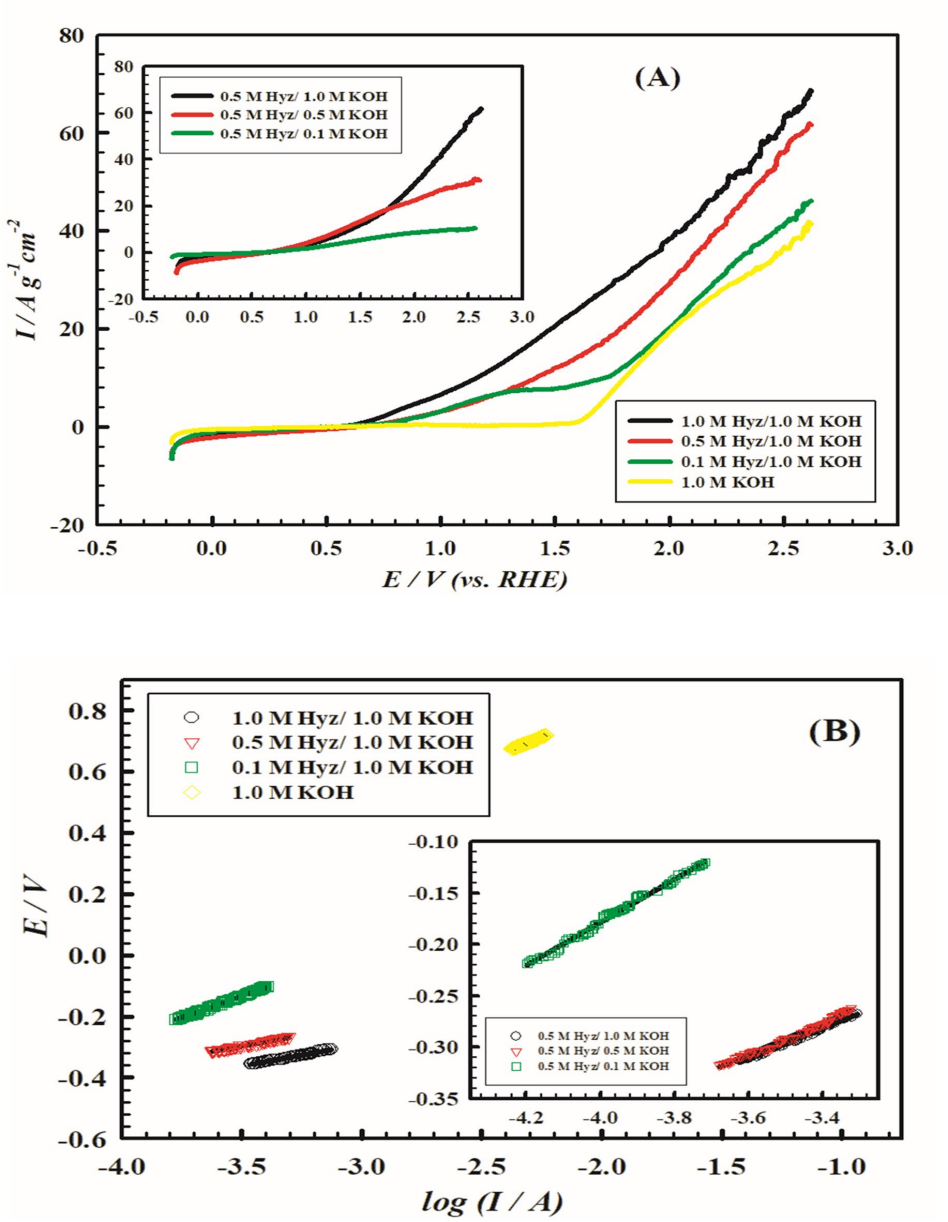
(**A**) LSV curves of electrode VI (PMTPEDOTNiOCPE) in 1.0 M KOH in presence of different concentrations of Hyz (0.1, 0.5 and 1.0 M). Inset: LSV curves of electrode VI in KOH with different concntrations (0.1, 0.5, and 1.0 M) in presence of 0.5 M Hyz. Scan rate 50 mV s^− 1^. (**B**) Tafel relations for OER at electrode VI in 1.0 M KOH in presence of different concentrations of Hyz (0.1, 0.5 and 1.0 M). Inset: Tafel relations for OER at electrode VI in KOH with different concntrations (0.1, 0.5, and 1.0 M) in presence of 0.5 M Hyz; scan rate 1.0 mV s^− 1^.

Electrolyte concentration is a crucial factor affecting the electrocatalytic process rate. The effect of using different concentrations of KOH in presence of 0.5 M Hyz on the OER kinetics is considered and illustrated in Fig. 7 (A) (inset). For all KOH concentrations, the OER onset potential is almost the same. The OER rate is enhanced by increasing the concentration of KOH.

On the other hand, the effect of using different concentrations of Hyz in basic medium on the OER rate is examined and illustrated in Fig. 7 (A). By increasing Hyz concentration, the OER onset potential is shifted to less positive values and the OER rate is enhanced.

The electrochemical performance of electrode VI in the presence of different concentrations of Hyz in 1.0 M KOH and in presence of different concentrations of KOH in presence of 0.5 M Hyz is probed by comparing their Tafel behaviors (Fig. 7 (B) and the inset, respectively). The Tafel slopes assumed from Fig. 7 (B) for electrode VI in 1.0 M KOH, 0.1 M Hyz/1.0 M KOH, 0.5 M Hyz/1.0 M KOH, and 1.0 M Hyz/1.0 M KOH are 310, 286, 147 and 144 mV.dec^− 1^, respectively. While the Tafel slopes assumed for electrode VI in 0.5 M Hyz/0.1 M KOH, 0.5 M Hyz/0.5 M KOH, and 0.5 M Hyz/1.0 M KOH are 209, 155, and 147 mV.dec^− 1^, respectively. The values of α_a_ and *J*_*°*_ for HyzOR at electrode VI in 0.5 M Hyz/(0.1, 0.5, 1.0 M) KOH and (0.1, 0.5, 1.0 M) Hyz/1.0 M KOH are summarized in Table 3. The values of α_a_ and *J*_*°*_ are enhanced upon increasing the concentration of KOH and Hyz reflecting enhanced kinetics for the HyzOR.

**Fig. 8:**
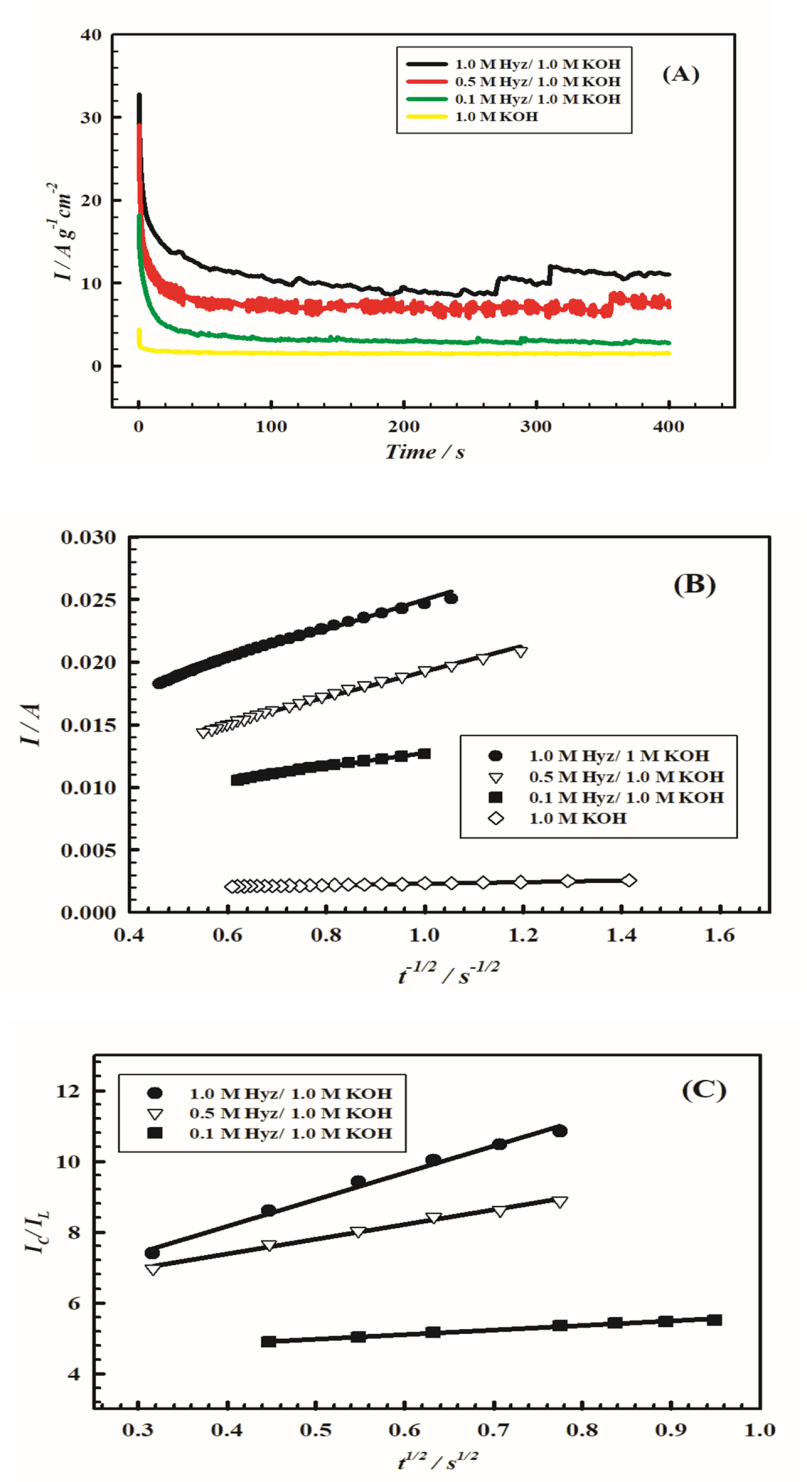
(**A**) Chronoamperograms over electrode VI (PMTPEDOTNiOCPE) in presence of different Hyz concentrations (0.0 M, 0.10 M, 0.50 M and 1 M in 1.0 M KOH). Applied potential is 630 mV. (**B**) Relations between catalytic oxidation currents (I_p_) and t ^-1/2^ at the electrode VI in presence of different Hyz concentrations (0.0 M, 0.10 M, 0.50 M and 1 M in 1.0 M KOH). (**C**) Relation between anodic current ratios in presence and absence of different concentrations of Hyz (0.0 M, 0.10 M, 0.50 M and 1 M in 1.0 M KOH) vs. t ^½^ at the electrode VI.

Figure 8 (A) shows the chronoamperograms for electrode VI in different Hyz concentrations compared to 1.0 M KOH. Constant potential values are applied and determined from the corresponding onset potentials of OER for each Hyz concentration. A fast depletion of current with time is observed in the first few seconds reaching a semi-steady value after 100 s limit. Considerable variations within the first few seconds occur at the surface of the electrode affecting different kinetic parameters.

**Table 3:** Exchange current density and charge transfer coefficient calculated from Tafel plots at electrode VI (PMTPEDOTNiOCPE) in different concentrations of Hyz and KOH.

Concentration Hyz/KOH	Charge transfer coefficient α_a_	Exchange current density j_o_ (A.cm^− 2^)
0.5 M Hyz/0.1 M KOH	0.929	2.20 × 10^− 3^
0.5 M Hyz/0.5 M KOH	0.905	7.48 × 10^− 2^
0.5 M Hyz/1.0 M KOH	0.900	1.02 × 10^− 1^
0.1 M Hyz/1.0 M KOH	0.948	2.82 × 10^− 3^
1.0 M Hyz/1.0 M KOH	0.897	3.20 × 10^− 1^
1.0 M KOH	0.952	8.73 × 10^− 5^

A plot of *I*_p_ versus *t*^− 1/2^ is shown in Fig. 8 (B) according to Eq. (11) to calculate the *D* values. Table 4 summarizes the *D* values in presence of different concentrations of Hyz in 1.0 M KOH at electrode VI. In addition, linear relations (Eq. 12) are obtained from the plots of the ratio of anodic current in presence and absence of different concentrations of Hyz in 1.0 M KOH vs. *t*^1/2^ as shown in Fig. 8 (C). The apparent rate constant *k* values are relatively high and increase with Hyz concentration reflecting effective charge transfer kinetics (Table 4).

As stated in Sect. 3.3.3, each of the constituents of the electrocatalyst contributes to the enhancement of both the diffusion coefficients and the corresponding rate constants. The two major factors are the intrinsic properties of each constituent and the relative increase in the electroactive surface area. Thus, for the mixture used of 1.5% PMT-PEDOT the electrical conductance is about 10^− 2^ S/cm that is reflected on better charge transfer throughout the electrocatalyst composite. NiO nanoparticles are known good electrocatalyst for OER in alkaline medium and the increase in the surface area increases the concentration of the reacting electrolyte at its surface. This synergistic contribution of the electrocatalyst composition leads to the observed enhanced reaction rate and lowering in the overpotential for the OER.

**Table 4:** The diffusion coefficient, *D*, and catalytic rate constant, *k*, for HyzOR-OER using different concentrations of Hyz/ 1.0 M KOH over electrode VI (PMTPEDOTNiOCPE).

Concentration Hyz/KOH	D / cm^2^.s^− 1^	k / cm^3^.mol^− 1^.s^− 1^
0.1 M Hyz/1.0 M KOH	6.08 × 10^− 7^	5.11 × 10^3^
0.5 M Hyz/1.0 M KOH	8.13 × 10^− 8^	1.08 × 10^4^
1.0 M Hyz/1.0 M KOH	2.88 × 10^− 8^	1.81 × 10^4^
1.0 M KOH	8.05 × 10^− 11^	….

#### Exhaustive electrolysis at PMTPEDOTNiOCPE

**Fig. 9:**
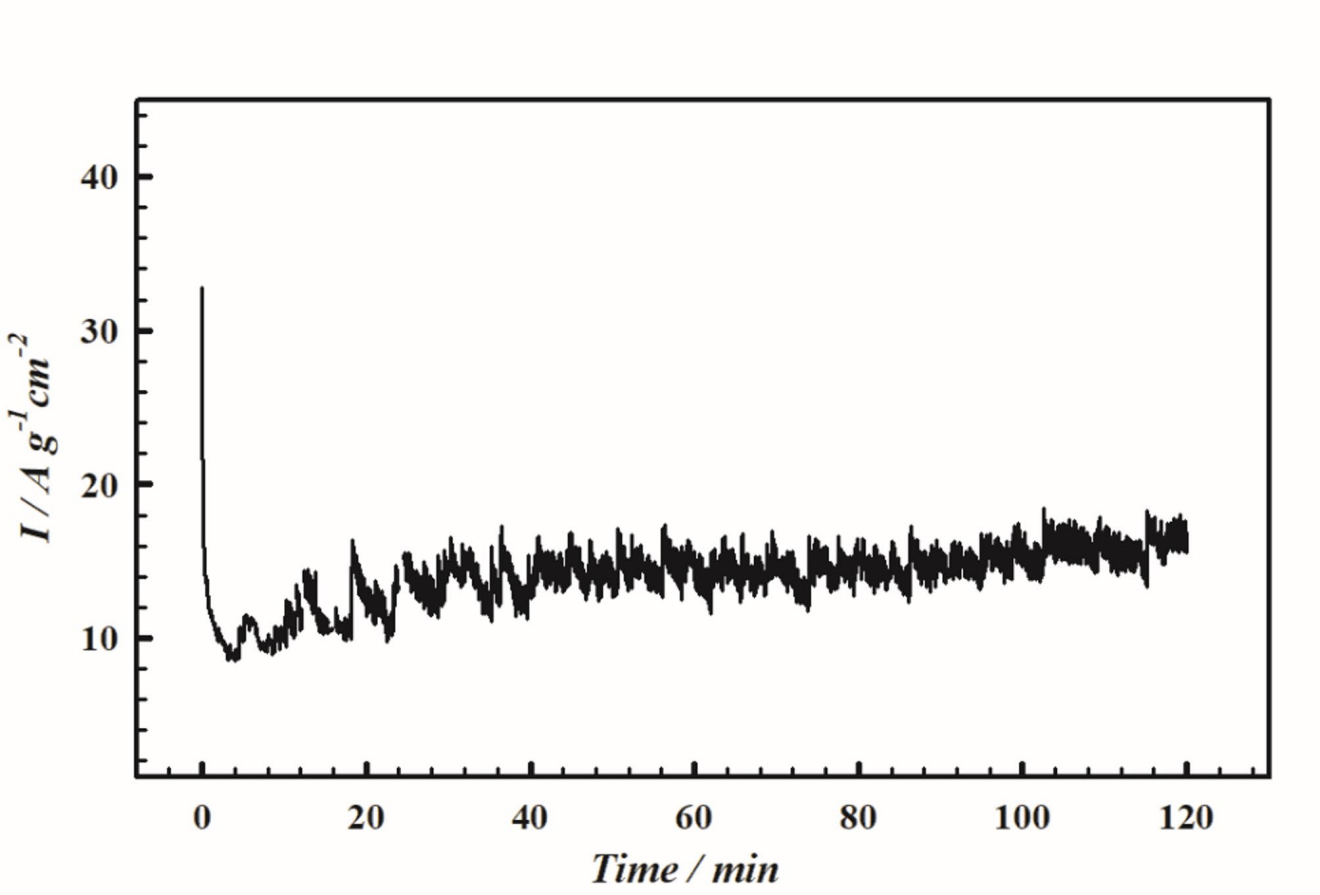
Chronoamperogram curve of electrode VI (PMTPEDOTNiOCPE) in 1.0 M Hyz/1.0 M KOH at 630 mV for two hours.

Subjecting the proposed electrode VI (PMTPEDOTNiOCPE) to exhaustive electrolysis is a crucial point to examine its electrochemical stability. This can be achieved using chronoamperometry in 1.0 M Hyz/1.0 M KOH for two hours at 630 mV (Fig. 9). The chronoamperogram indicates that the proposed surface shows a stable current response during exhaustive electrolysis.

#### Temperature effect on the catalytic performance of PMTPEDOTNiOCPE toward the HyzOR-OER process

Temperature is a crucial parameter affecting the performance of different catalysts in the electrochemical conversion processes. Therefore, electrode VI (PMTPEDOTNiOCPE) is tested at different temperatures (10–50 °C) in 1.0 M Hyz/1.0 M KOH for the HyzOR-OER using linear sweep voltammetry at scan rate of 50 mV.s^− 1^. There is an obvious decrease in the onset potential for the conversion process and an increase in the current-voltage relation slope following the onset potential. A certain potential value is selected for all temperatures studied and the corresponding current values are determined. The activation energy is calculated from Arrhenius relation according to the following equation (Eq. 13) ^20^;

13 $$\:Log\:{j}_{^\circ\:}=-2.303\:{E}_{a}/RT+Log\:A$$

**Fig. 10:**
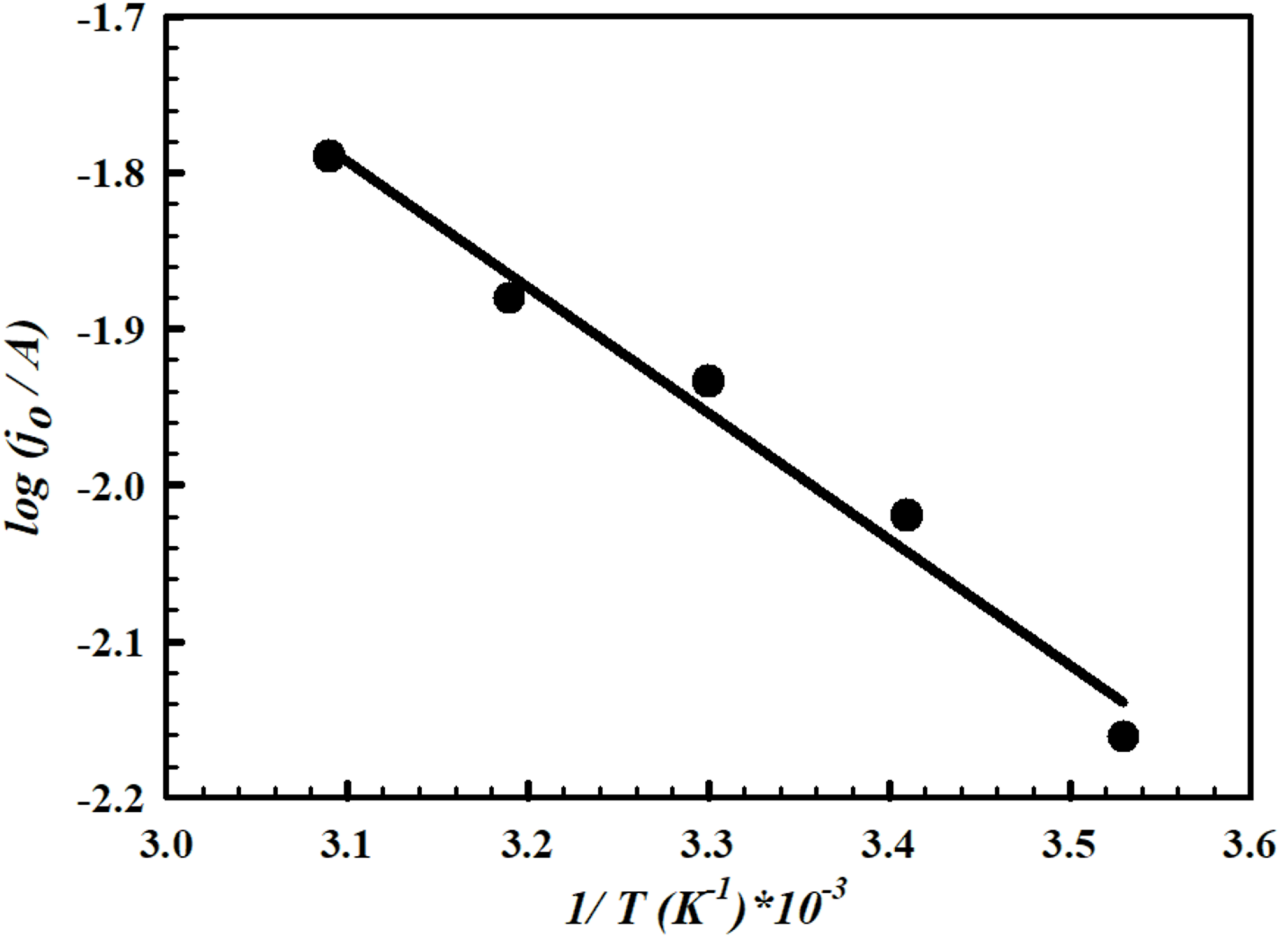
Arrhenius plot of log (anodic peak currents A) vs. 1/T (K^− 1^) in 1.0 M Hyz /1.0 M KOH at electrode VI (PMTPEDOTNiOCPE).

Where* j*_o_ is the anodic current (A), *E*_a_ is the activation energy (J.mol^− 1^), *T* is the absolute temperature (K), *R* is the universal gas constant (8.314 J.K^− 1^.mol^− 1^) and *A* is a pre-exponent factor. The activation energy value calculated from the above equation is 2.90 kJ.mol^− 1^. Figure 10 shows the Arrhenius relation between (*log j*_o_) versus (*1/T*) at different temperature values (10–50 °C). The value of *E*_a_ is relatively low ensuring the facilitation of HyzOR-OER at electrode VI catalyst. Some literature reports relatively higher values of activation energy for other electrochemical conversion processes reflecting the advantage of the proposed catalyst for HyzOR-OER^20,67^.

#### Effect of using POTPEDOTNiOCPE on hydrazine-assisted water splitting

**Fig. 11:**
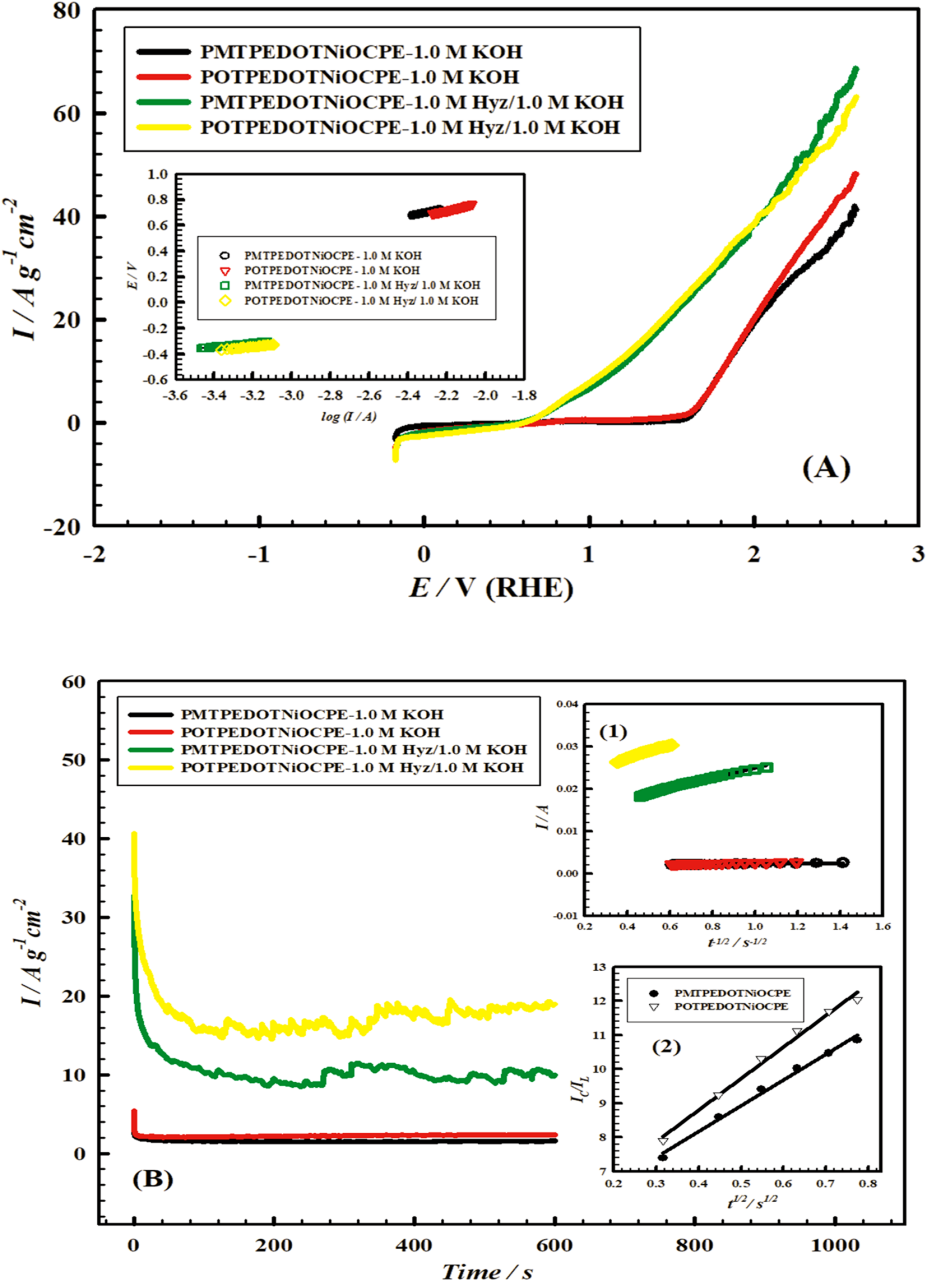
(**A**) Linear sweep voltammogram curves of electrode VI (PMTPEDOTNiOCPE) and electrode VII (POTPEDOTNiOCPE) in 1.0 M KOH in presence and absence of 1.0 M Hyz. Scan rate 50 mV s^− 1^. Inset: Tafel relations for OER of electrodes VI and VII in 1.0 M KOH in presence and absence of 1.0 M Hyz; scan rate 1.0 mV s^− 1^. (**B**) Chronoamperograms over electrodes VI and VII in 1.0 M KOH in presence and absence of 1.0 M Hyz. Applied potential is 630 mV. Inset (1): Relations between catalytic oxidation currents (I_p_) and t^-1/2^ at electrodes VI and VII in 1.0 M KOH in presence and absence of 1.0 M Hyz. Inset (2): Relation between anodic current ratios and t^½^ at electrodes VI and VII in 1.0 M KOH in presence and absence of 1.0 M Hyz.

The influence of using poly (o-toluidine), POT instead of PMT in the composite on the hydrazine-assisted water splitting is investigated. Figure 11 (A) shows the LSVs of electrode VII (POTPEDOTNiOCPE) and electrode VI (PMTPEDOTNiOCPE) in 1.0 M Hyz/1.0 M KOH and in absence of Hyz. Figure 11 (A) (Inset) shows the Tafel plots for electrodes VII and VI in 1.0 M Hyz/1.0 M KOH and in Hyz-free solution (1.0 M KOH). The Tafel slopes assumed for electrode VII in 1.0 M Hyz/1.0 M KOH, and 1.0 M KOH are 147, and 362 mV current dec^− 1^, respectively. While the Tafel slopes assumed for electrode VI in 1.0 M Hyz/1.0 M KOH, and 1.0 M KOH are 144, and 310 mV.dec^− 1^, respectively. The values of α_a_ and *J*_*°*_ for OER for electrodes VII and VI in 1.0 M Hyz/1.0 M KOH and in absence of Hyz are summarized in Table 5. The values of α_a_ and *J*_*°*_ are enhanced upon using POT instead of PMT reflecting enhanced kinetics for the OER in presence and absence of 1.0 M Hyz.

Figure 11 (B) shows the chronoamperograms for electrodes VII and VI in 1.0 M Hyz/1.0 M KOH and in absence of Hyz. Figure 11 (B) (Insets 1 and 2) show the linear relations used to calculate the kinetic parameters, *D* and *k*, respectively. Table 6 summarizes the *D* and *k* values in presence and absence of 1.0 M Hyz at electrodes VII and VI. Electrode VII shows higher values of *D* and *k* compared to electrode VI. This may be attributed to higher conductivity and electrocatalytic activity of POT (poly(2-methylaniline)) compared to PMT (poly(3-methylaniline))^68–70^.

**Table 5:** Exchange current density and charge transfer coefficient calculated from Tafel plots for electrode VI (PMTPEDOTNiOCPE) and electrode VII (POTPEDOTNiOCPE) in 1.0 M KOH and 1.0 M Hyz/1.0 M KOH.

Surface	1.0 M KOH		1.0 M Hyz/ 1.0 M KOH	
	α_a_	j_o_ (A cm^− 2^)	α_a_	j_o_ (A cm^− 2^)
VI (PMTPEDOTNiOCPE)	0.952	8.73 × 10^− 5^	0.897	3.20 × 10^− 1^
VII (POTPEDOTNiOCPE)	0.959	2.09 × 10^− 4^	0.899	4.19 × 10^− 1^

**Table 6:** The diffusion coefficient, *D*, and catalytic rate constant, *k*, for HyzOR-OER for electrode VI (PMTPEDOTNiOCPE) and electrode VII (POTPEDOTNiOCPE) in 1.0 M KOH and 1.0 M Hyz/1.0 M KOH.

Surface	1.0 M KOH		1.0 M Hyz/ 1.0 M KOH	
	D / cm^2^ s^− 1^	k / cm^3^ mol^− 1^ s^− 1^	D / cm^2^ s^− 1^	k / cm^3^ mol^− 1^ s^− 1^
VI (PMTPEDOTNiOCPE)	8.05 × 10^− 11^	…	2.88 × 10^− 8^	1.81 × 10^4^
VII (POTPEDOTNiOCPE)	1.22 × 10^− 10^	…	5.47 × 10^− 8^	2.71 × 10^4^

## Conclusion

In summary, energy-efficient electrocatalyst is needed to boost the electrochemical synthesis of H_2_. The combination of HER and HyzOR requiring lower thermodynamic oxidation potential is more cost-effective than the use of OER for large scale H_2_ production. A single-step strategy for NiO nano-catalyst mixed with a conducting polymer mixture of poly (m-toluidine) and poly (3,4-ethylenedioxythiophene) modified carbon paste electrode is reported here for electrochemical water splitting in the presence of hydrazine. Hydrazine oxidation supports the OER. The OER occurs in 1.0 M KOH at an onset potential of + 0.626 V (overpotential= ‒0.604 V) (RHE) in presence of 1.0 M Hyz with a specific current density of 0.903 A.g^− 1^.cm^− 2^. While the OER onset potential in absence of Hyz is + 1.605 V (overpotential = + 0.375 V) (RHE) with a specific current density of 0.380 A.g^− 1^.cm^− 2^. A shift in the potential of 0.979 V is observed in 1.0 M Hyz/1.0 M KOH compared to Hyz-free solution (KOH only). Each component of the presented surface has a role in the improvement of overall water splitting. PMTPEDOTNiOCPE exhibits good stability for exhaustive electrolysis applications with no decrease in the current response through chronoamperometry in 1.0 M Hyz/ 1.0 M KOH for two hours. The activation energy value is 2.90 kJ.mol^− 1^ which is lower than that reported elsewhere reflecting the ease electrocatalytic process of HyzOR-OER at the presented surface. Higher values of diffusion coefficient and rate constant are achieved at the proposed surface compared to its individual components ascertaining the interactive collaboration between the different modifiers of the surface.

## Electronic supplementary material

Below is the link to the electronic supplementary material.

Supplementary Material 1

## Data Availability

All data generated or analysed during this study are included in this published article [and its supplementary information files.
