# Supplementary material for: NiO@conducting polymer electrocatalyst for hydrazine-assisted oxygen evolution reaction through water splitting
Source: Sci Rep. 2025 Jul 25;15:27169. doi: 10.1038/s41598-025-09480-3 (PMC12297418; doi:10.1038/s41598-025-09480-3)
Supplement: Supplementary file 1 — Supplementary Material 1 [file 41598_2025_9480_MOESM1_ESM.docx]

**NiO@conducting polymer electrocatalyst for hydrazine-assisted oxygen evolution reaction through water splitting**

Ekram H. El-Ads ^1,*^, Moshira M. Khalil^@^, Mahmoud A. Abd El-Ghaffar^2^, Ahmed Galal^1^

^1^Cairo University, Faculty of Science, Chemistry Department, Giza, Postal Code 12613, Egypt

^2^Polymers and Pigments Department, National Research Center, Cairo, Egypt

^*^Corresponding author. E-mail address: [ekram@sci.cu.edu.eg](mailto:ekram@sci.cu.edu.eg)

- In the supplementary file we provide nine Supplement Figures (1-9) which provide more details about the current work.

**Supplement Figure 1**


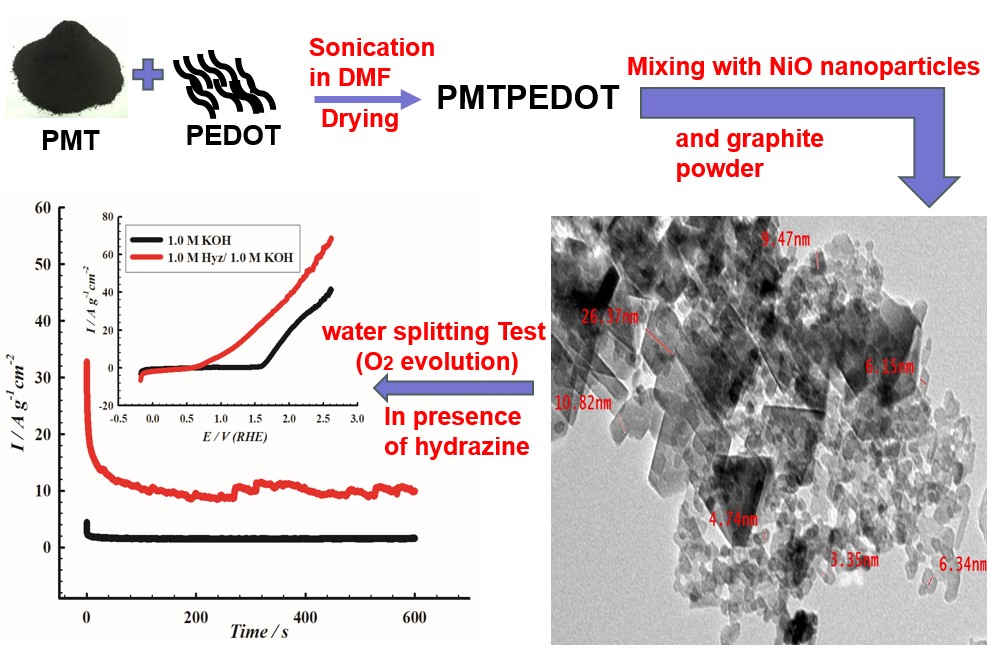


**Supplement Figure 1:** A graphical summary of the methodology and objective of this work.

**Supplement Figure 2**

| 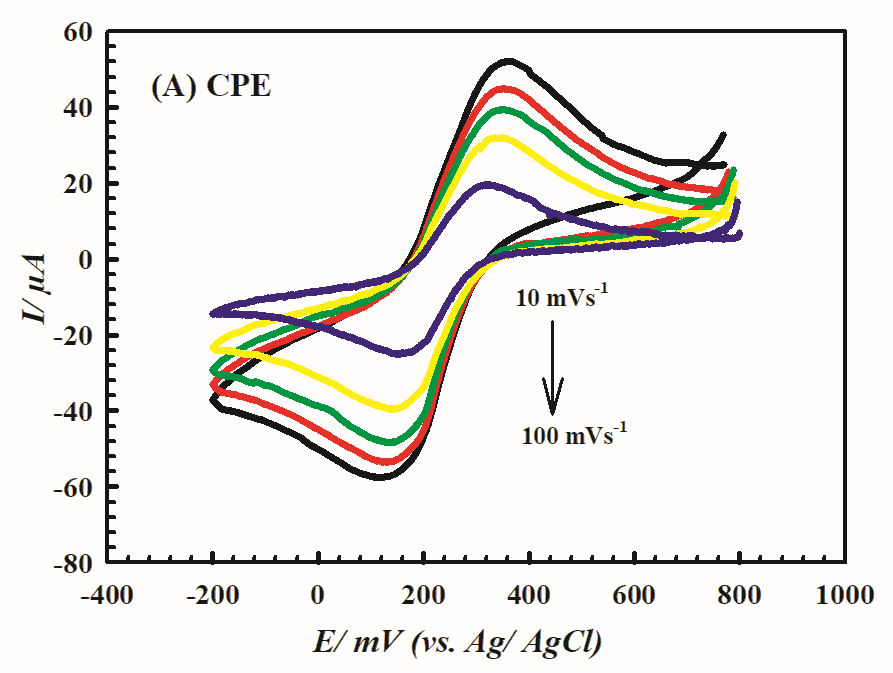 | 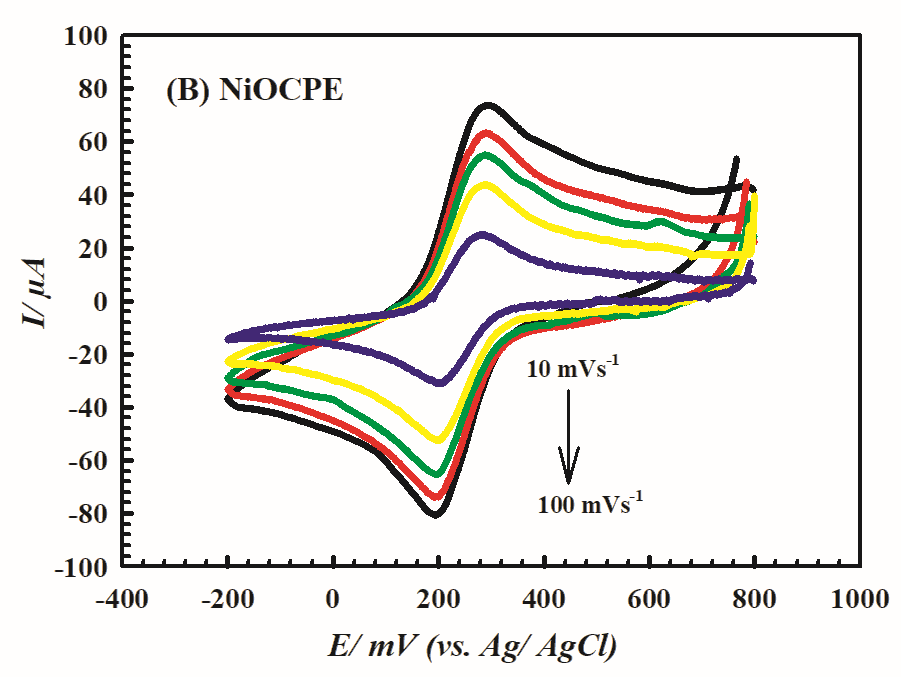 |
| --- | --- |
| 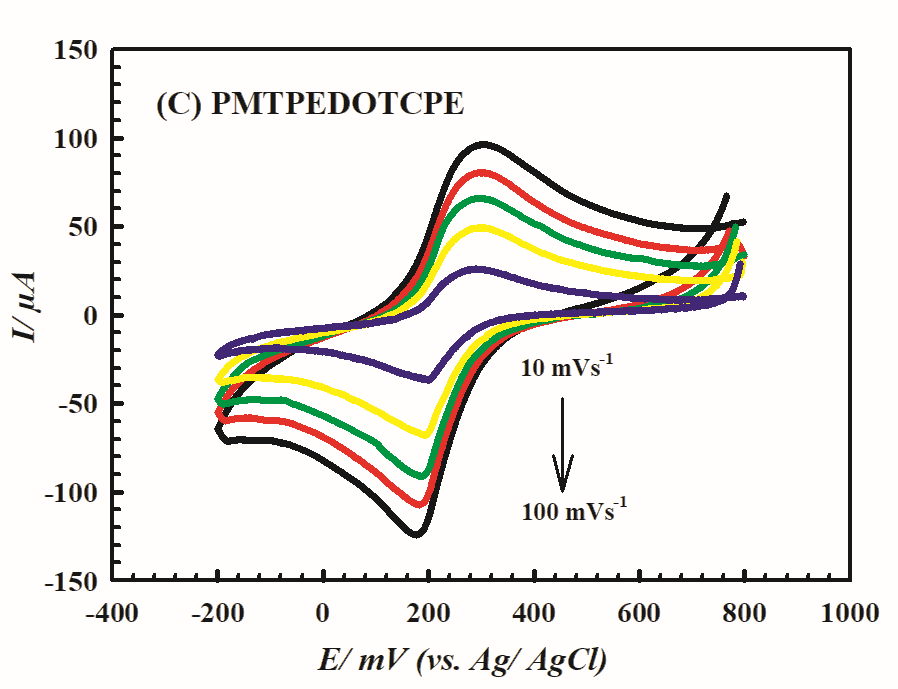 | 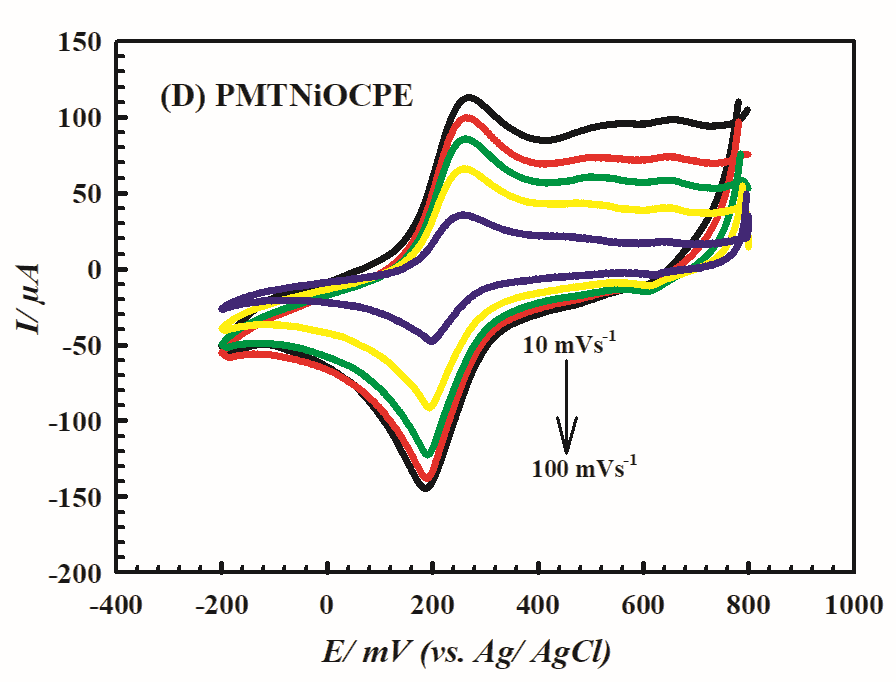 |
| 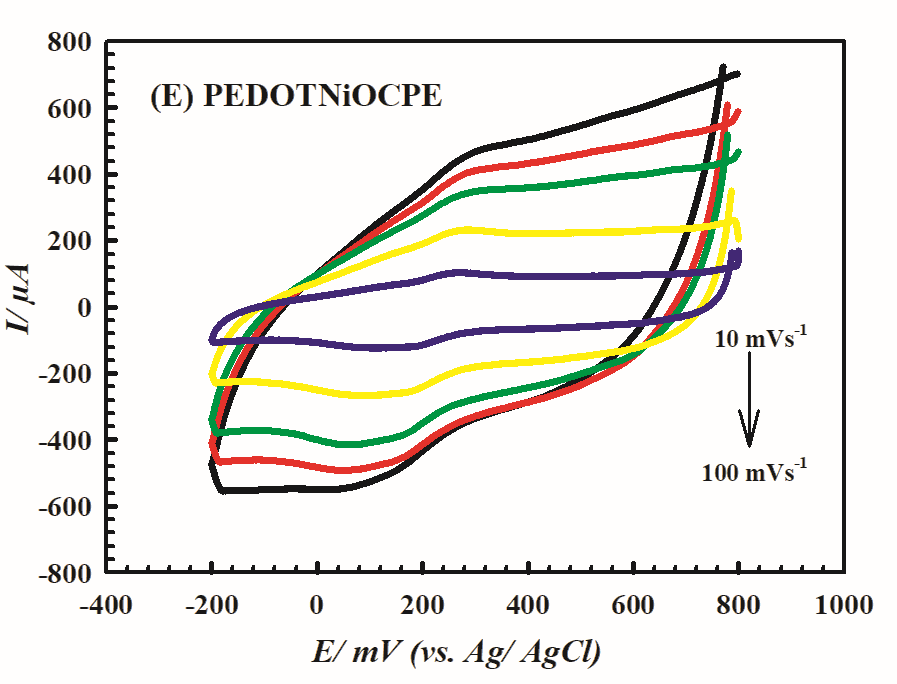 | 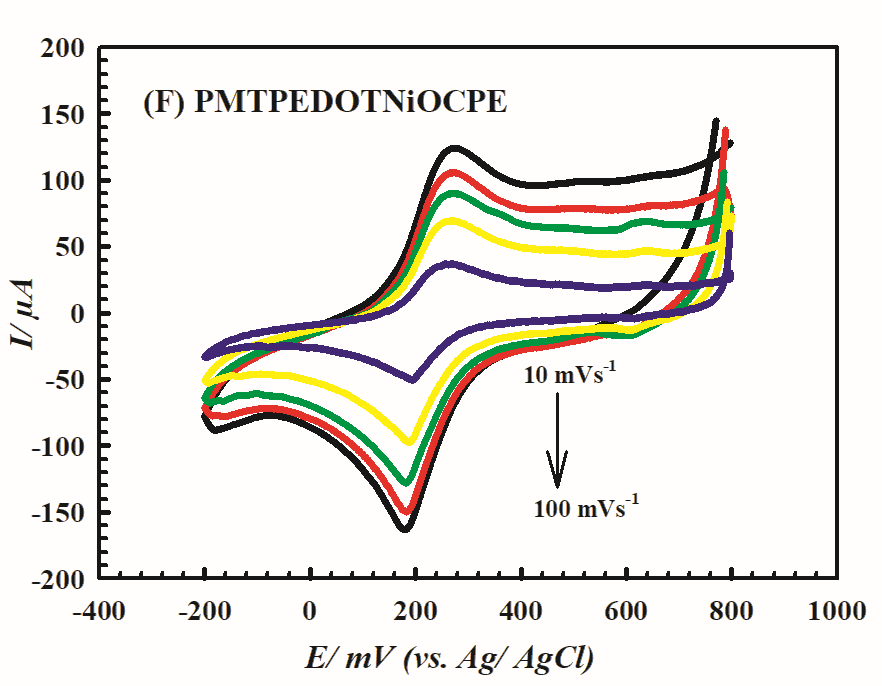 |
| 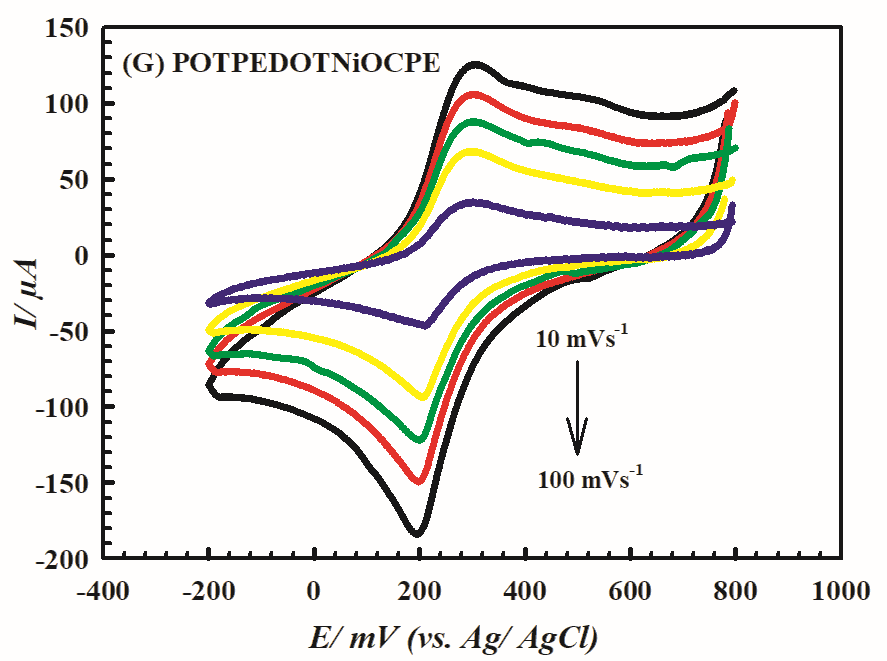 | |

**Supplement Figure 2:** CVs of (A) CPE, (B) NiOCPE, (C) PMTPEDOTCPE, (D) PMTNiOCPE, (E) PEDOTNiOCPE, (F) PMTPEDOTNiOCPE and (G) POTPEDOTNiOCPE in 1 mM K_3_Fe(CN)_6_ at different scan rate values (10 to 100 mVs^-1^).

The electro-active surface areas of the modified electrodes were determined by CV technique, using 1.0 mM K_3_Fe(CN)_6_. The surface areas were calculated using Randles-Sevick equation

I_p_ = 2.69×10^5^n^3/2^AD^1/2^C_o_ν^1/2^

For K_3_Fe(CN)_6_, n = 1, *D* = 7.6 × 10^−6^ cm^2^·s^−1^ and ν is the scan rate (10 to 100 mVs^-1^). A linear relationship was obtained between I_p_ and ν^1/2^ and the electro-active surface areas can be calculated from the slope.

**Supplement Figure 3**

| **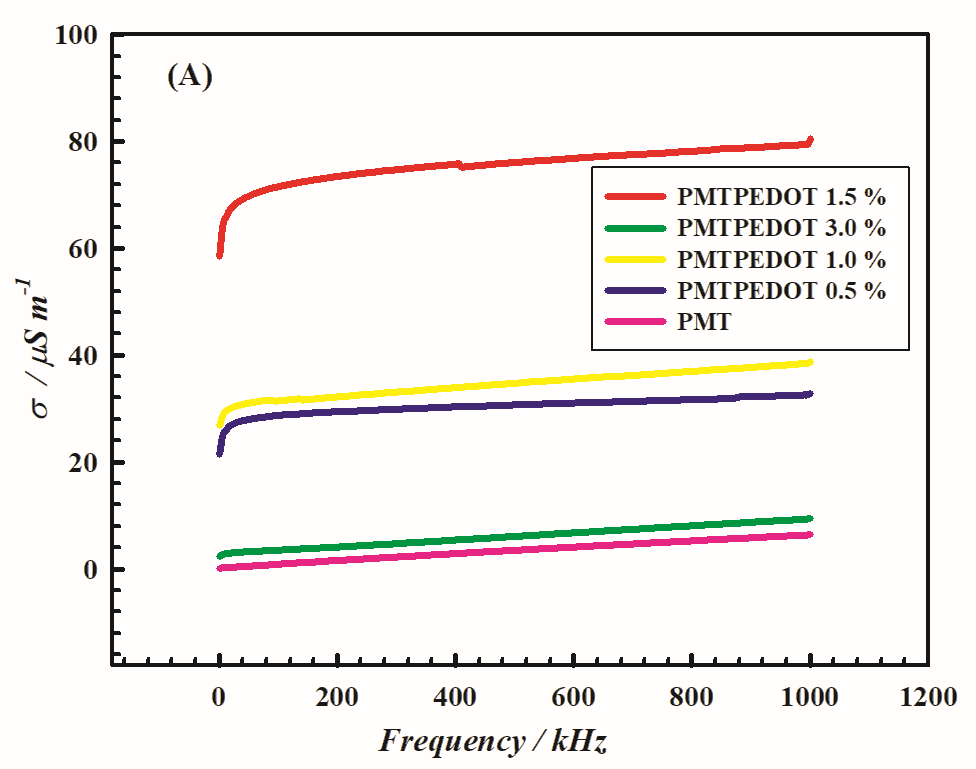** | **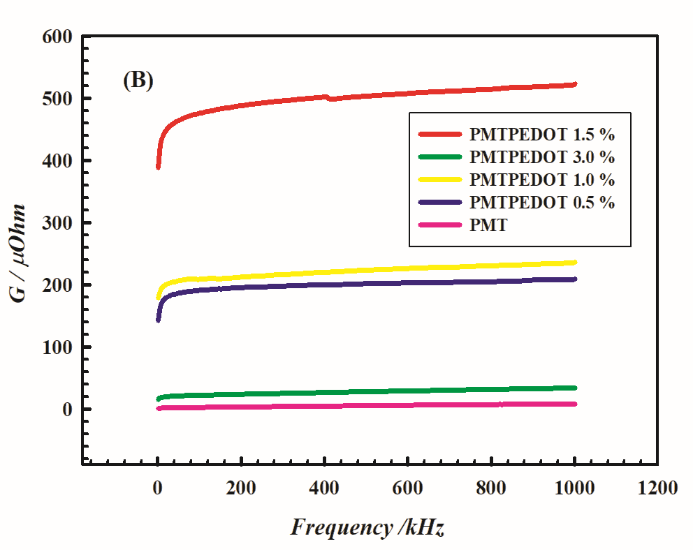** |
| --- | --- |
| **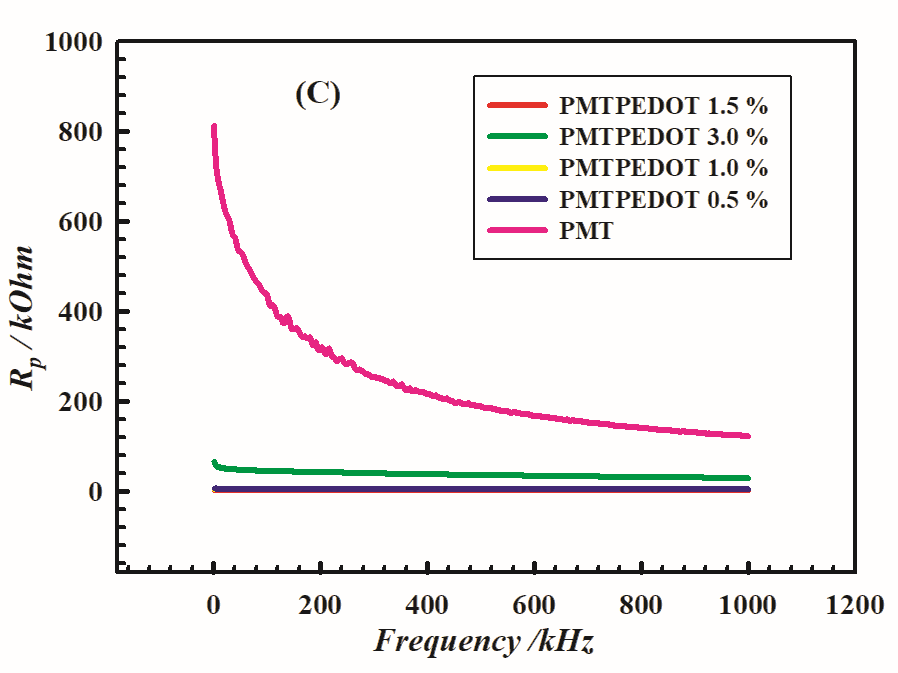** | **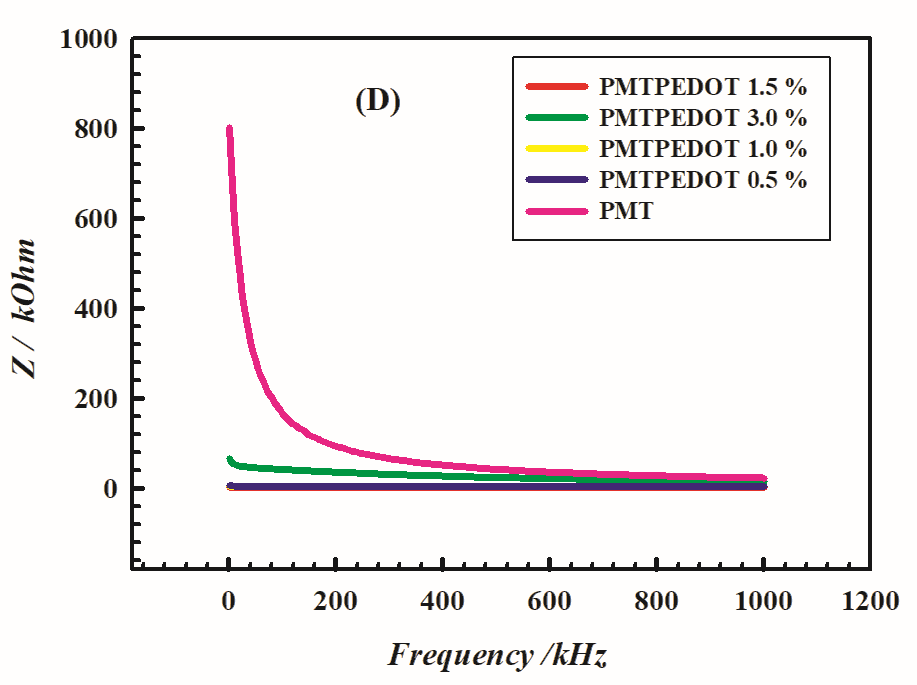** |
| **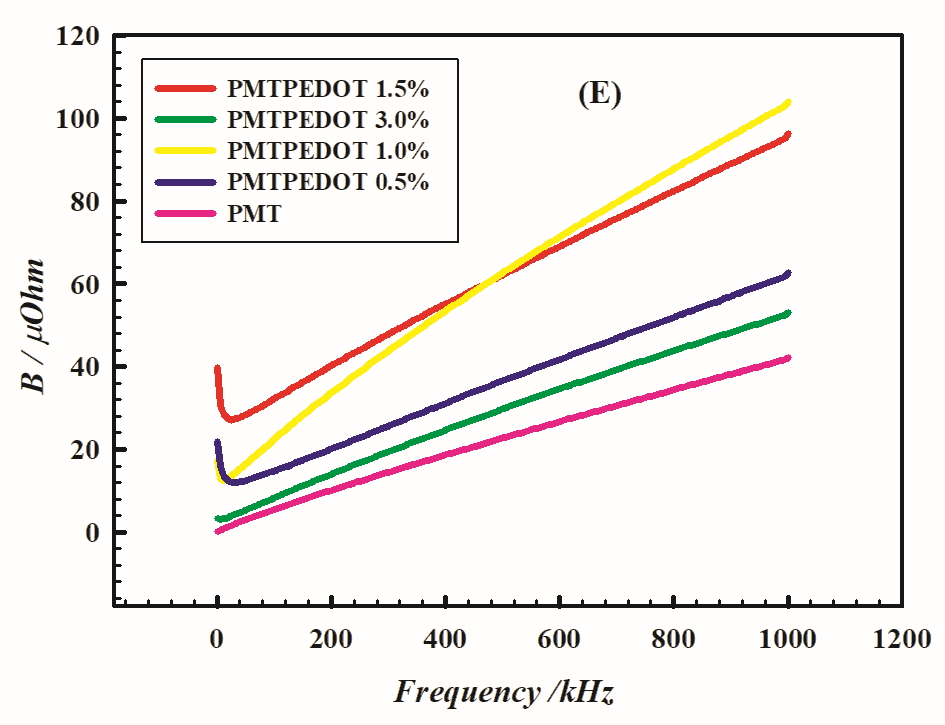** | |

**Supplement Figure 3:** Electrical properties of a series of PMTPEDOT mixture (PMT with different amounts of PEDOT (0.5%, 1%, 1.5% and 3%)) over the frequency range of 10^3^-10^6^ Hz.

**Supplement Figure 4**

| 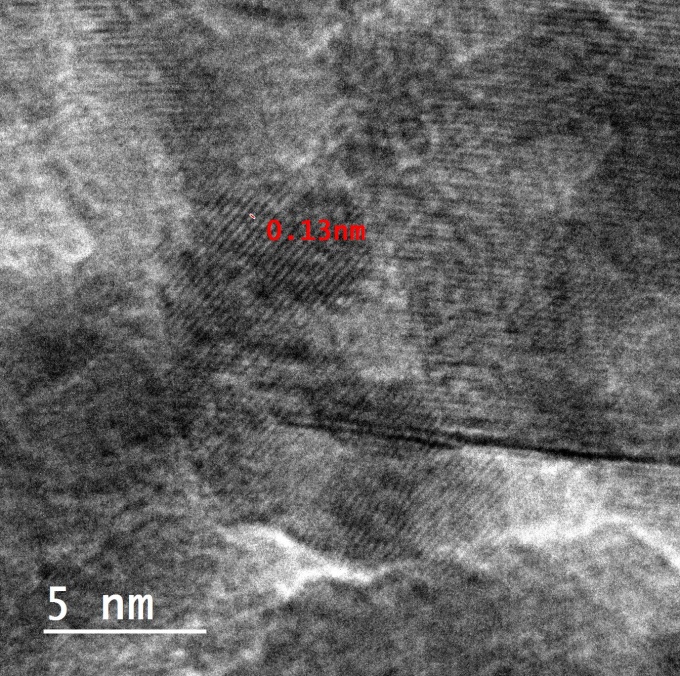  **(A)** | 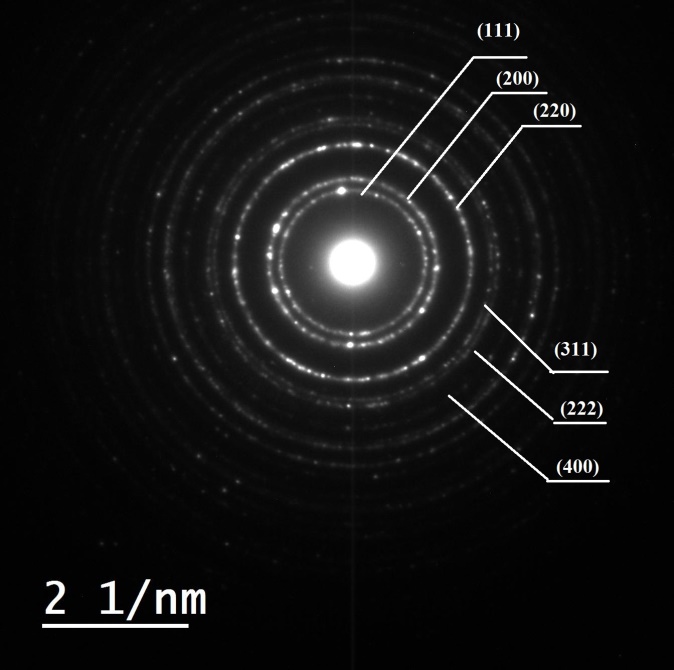  **(B)** |
| --- | --- |
| 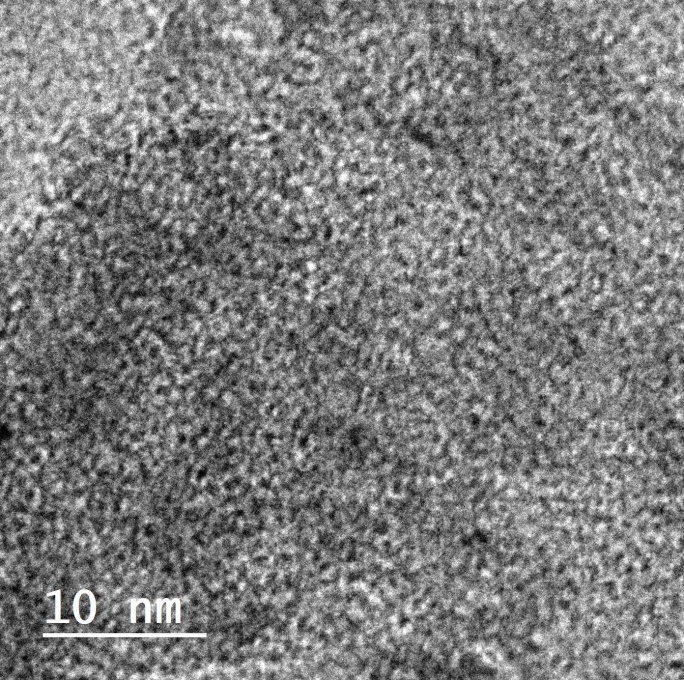  **(C)** | 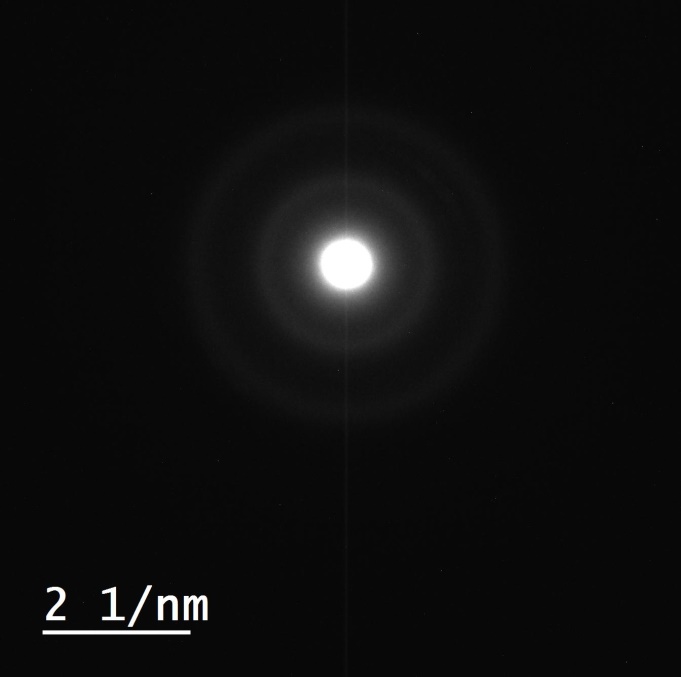  **(D)** |

**Supplement Figure 4:** HRTEM image of PMTPEDOTNiO including (A) NiO texture and (C) PMTPEDOT texture. SAED pattern of HRTEM of (B) NiO and (D) PMTPEDOT.

**Supplement Figure 5**

**
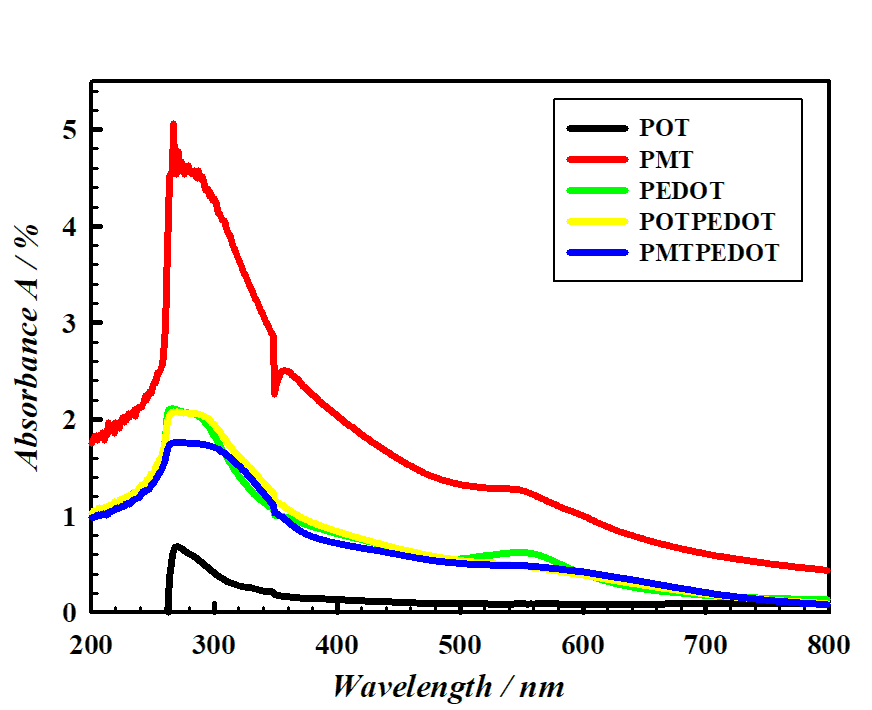
**

**Supplement Figure 5:** UV-vis absorption spectra of POT, PMT, PEDOT, POTPEDOT and PMTPEDOT.

**Supplement Figure 6**


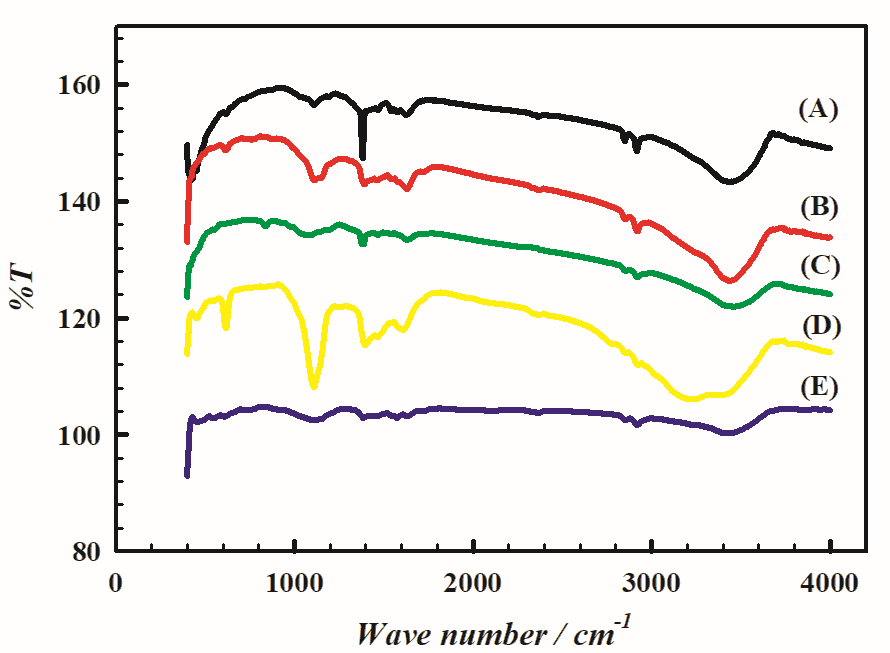


**Supplement Figure 6:** FTIR spectra of **(A)** NiO, **(B)** PMTNiO, **(C)** PEDOTNiO, **(D)** PMTPEDOTNiO, and **(E)** PMTPEDOTNiOCPE.

**Supplement Figure 7**

| **NiO**  **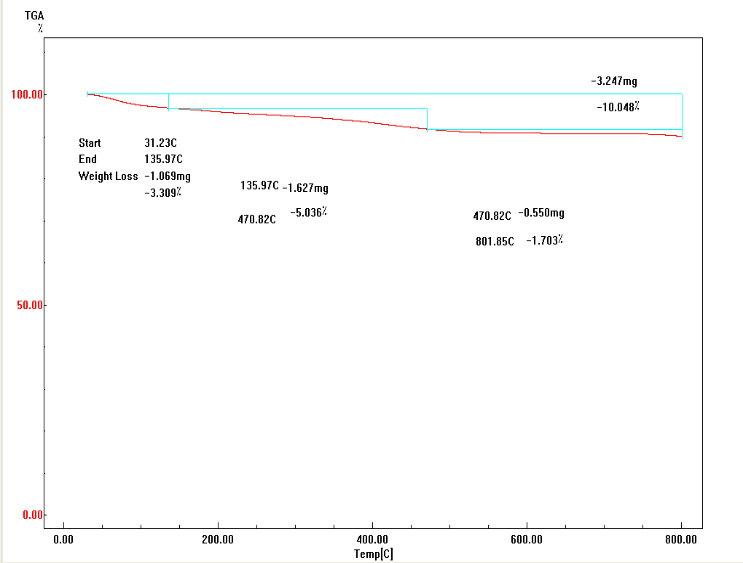** | **NiO**  **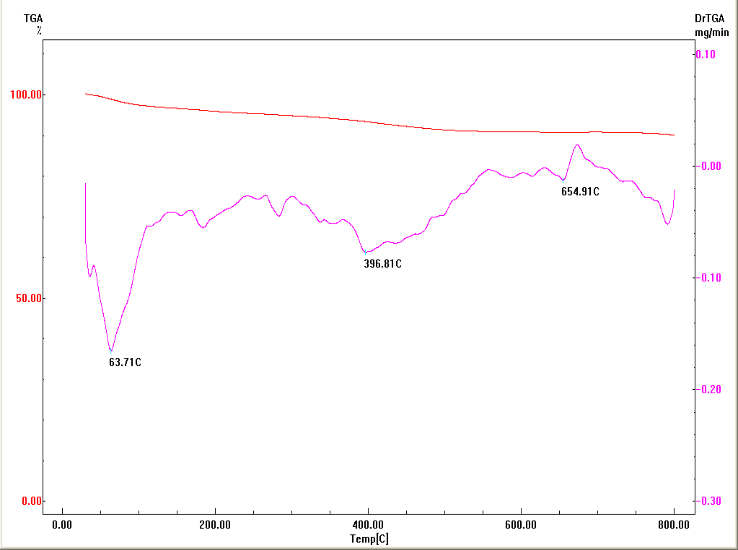** |
| --- | --- |
| **PMTNiO**  **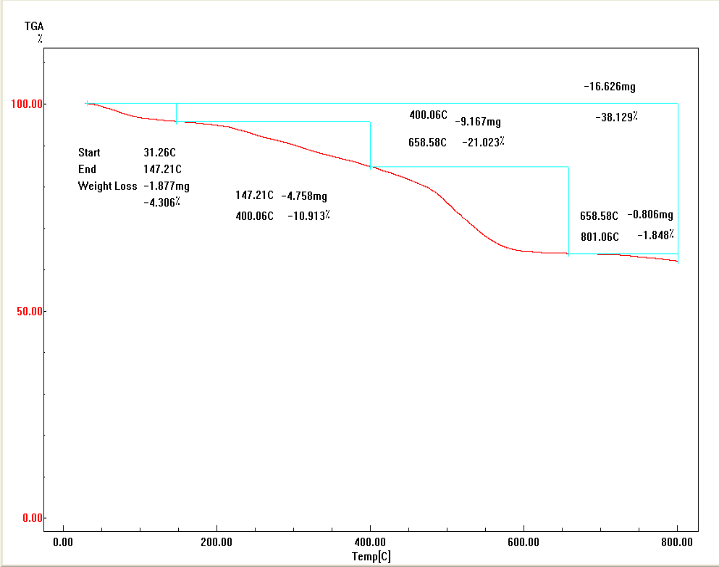** | **PMTNiO**  **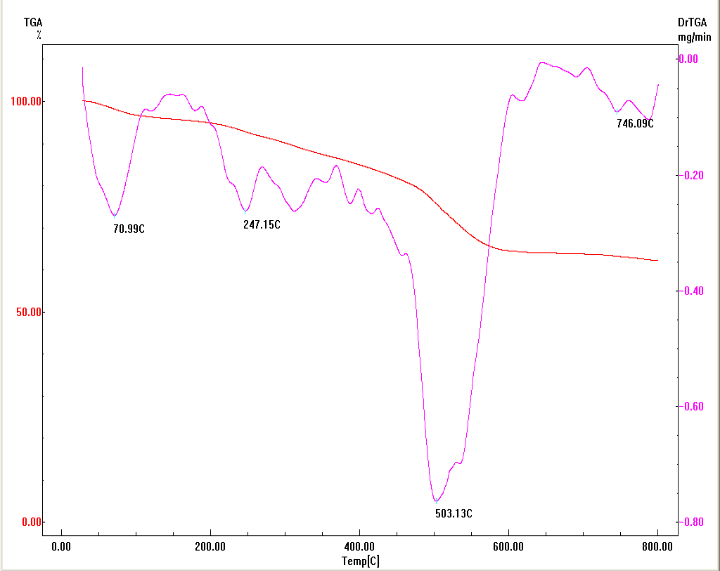** |
| **PEDOTNiO**  **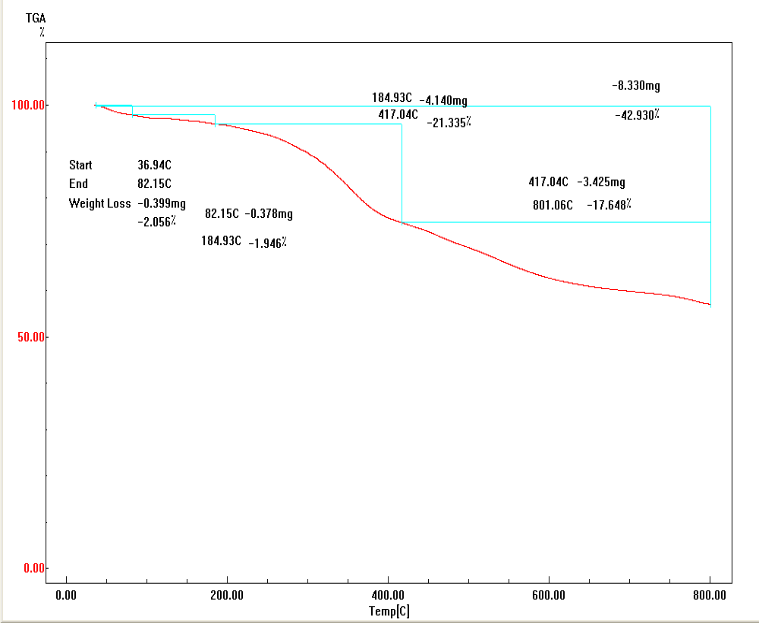** | **PEDOTNiO**  **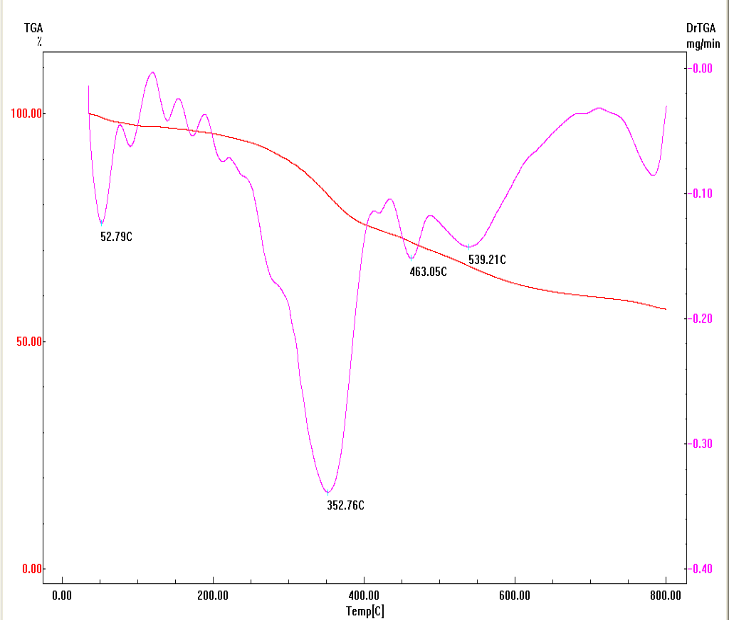** |
| **PMTPEDOTNiO**  **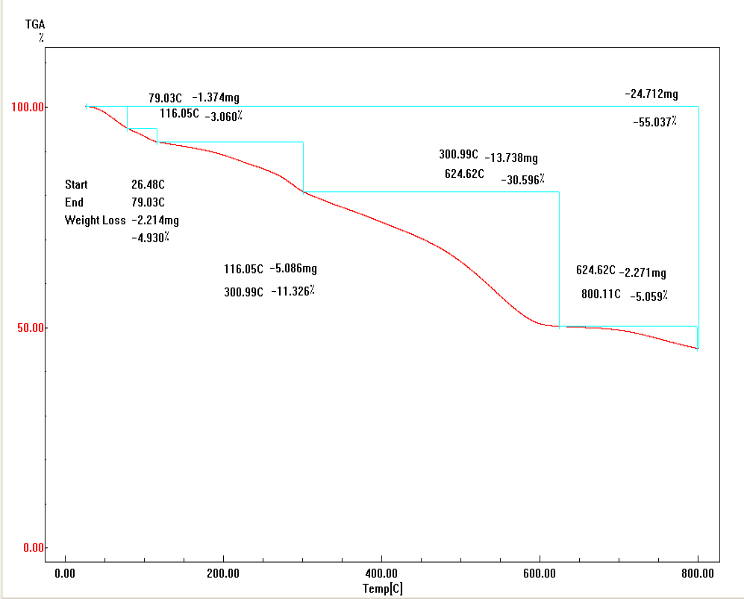** | **PMTPEDOTNiO**  **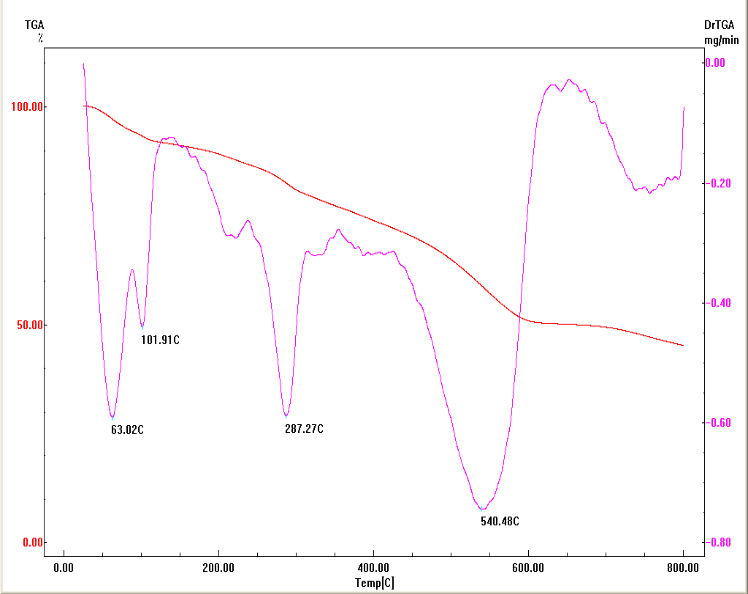** |
| **PMTPEDOTNiOCPE**  **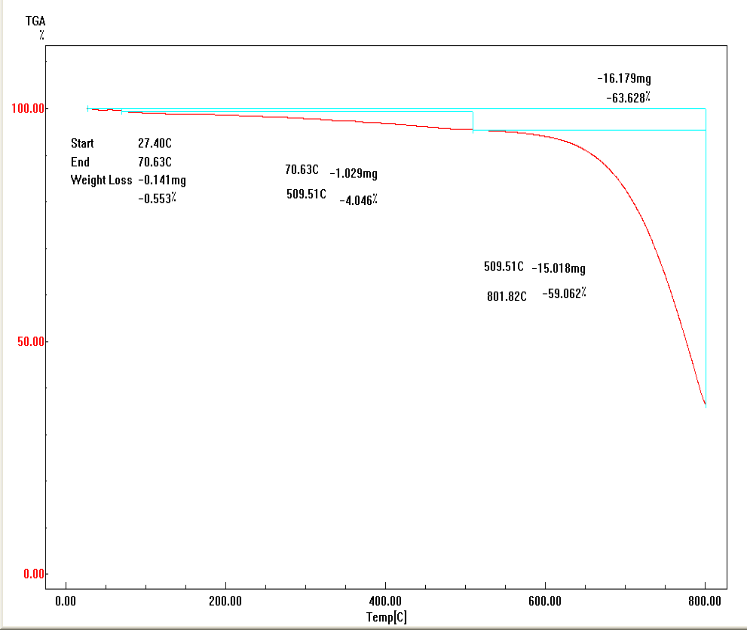** | **PMTPEDOTNiOCPE**  **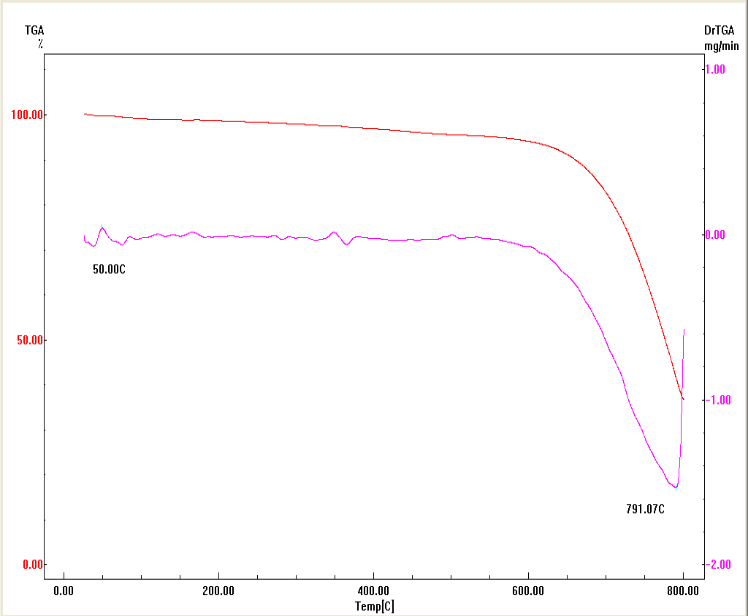** |

**Supplement Figure 7:** Thermal gravimetric analysis (TGA) and its differential (drTGA) of NiO, PMTNiO, PEDOTNiO, PMTPEDOTNiO, and PMTPEDOTNiOCPE.

**Supplement Figure 8**


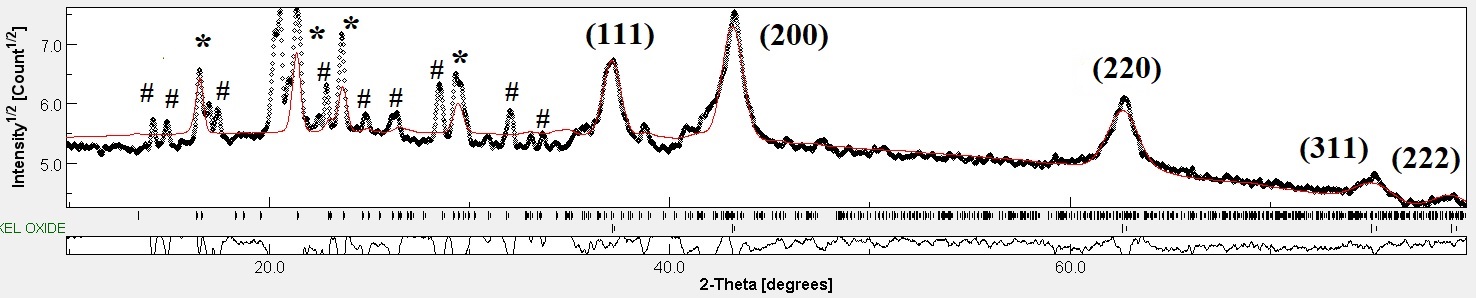


**Supplement Figure 8:** Rietveld refinement of the XRD of the composite PMTPEDOTNiO; the different phases of NiO are indicated, (*) represent the fitting of PEDOT diffraction pattern, (#) represent non-identified phase(s).

**Supplement Figure 9**

| 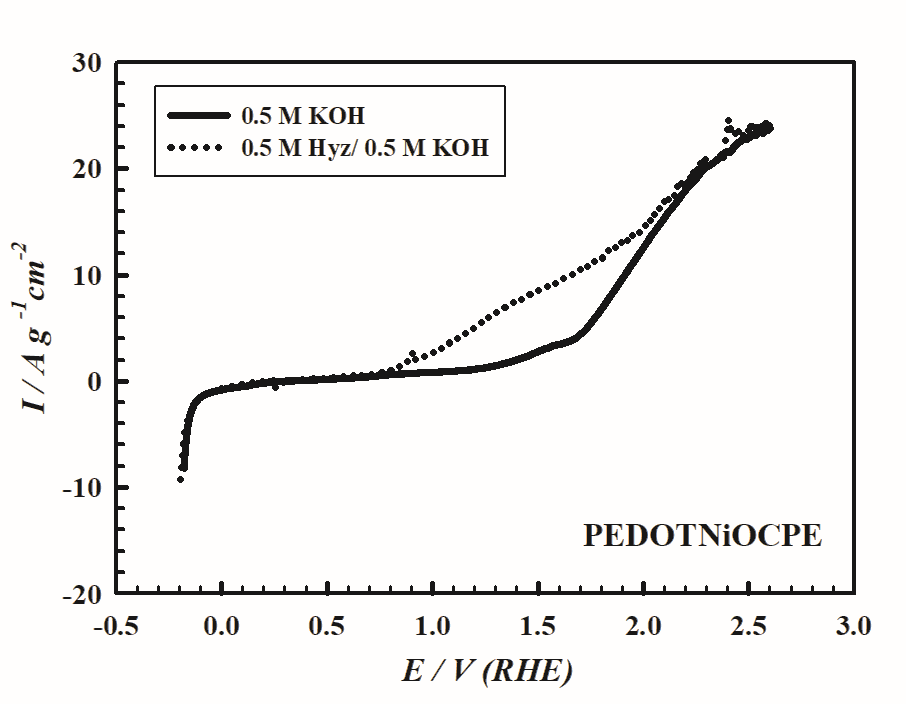 | 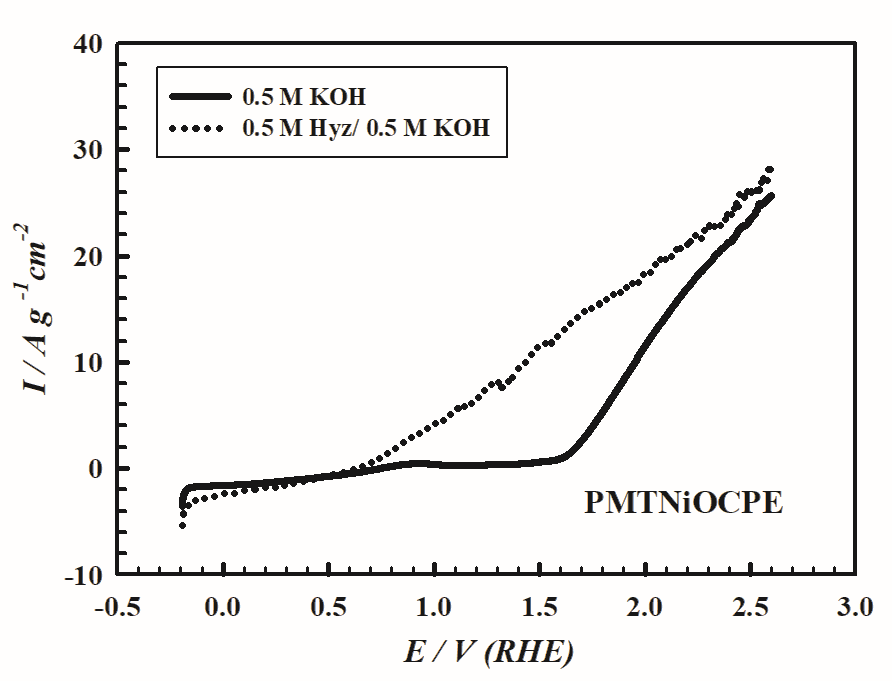 |
| --- | --- |
| 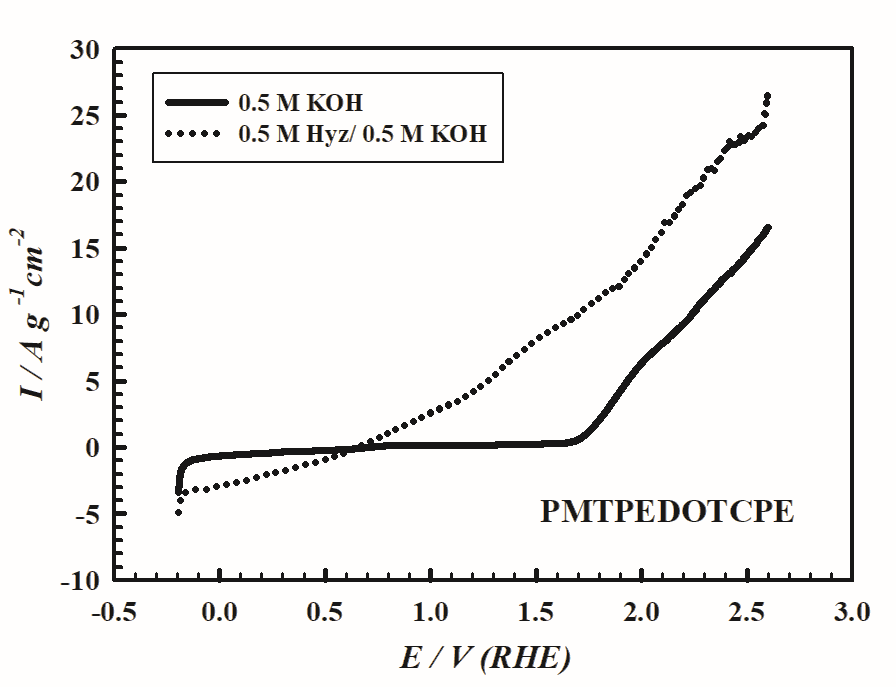 | 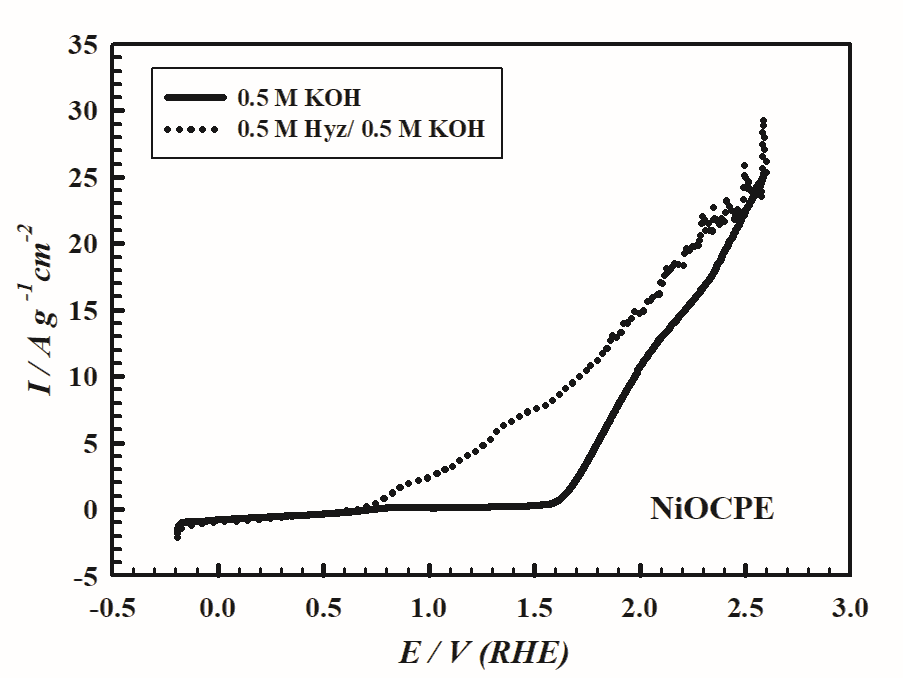 |
| 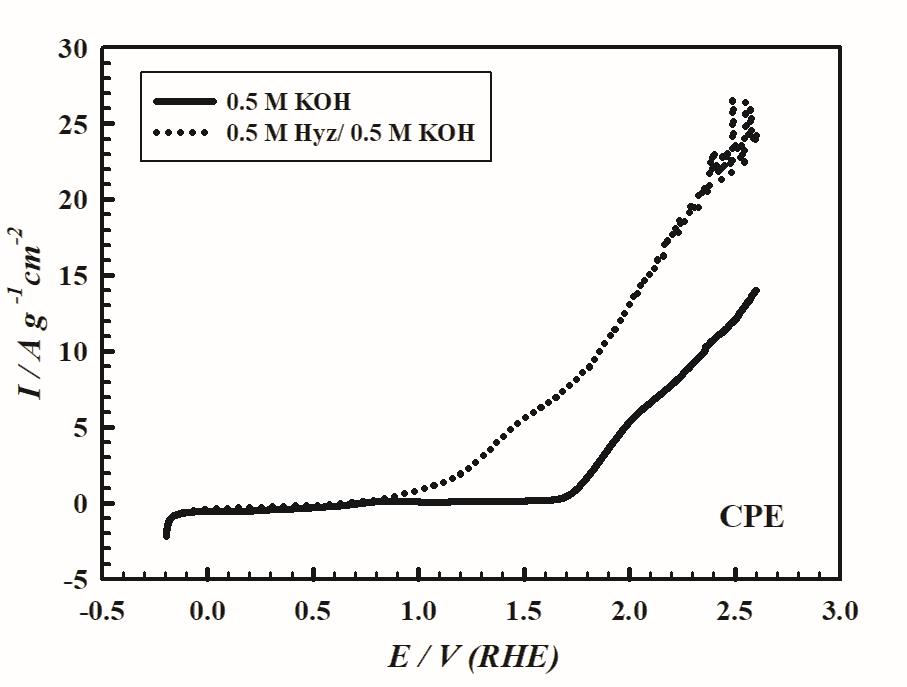 | |

**Supplement Figure 9:** Linear sweep voltammogram (LSV) curves of PEDOTNiOCPE, PMTNiOCPE, PMTPEDOTCPE, NiOCPE, and CPE in 0.5 M KOH in presence and absence of 0.5 M Hyz. Scan rate 50 mV.s^−1^.
